# Directed evolution of a stem-helix–targeting antibody enables MERS-CoV cross-neutralization through enhanced binding affinity

**DOI:** 10.1371/journal.ppat.1014393

**Published:** 2026-08-07

**Authors:** Panpan Zhou, Meng Yuan, Yuexiu Zhang, Oliver Limbo, Ge Song, Fangzhu Zhao, Hejun Liu, Wan-ting He, Tazio Capozzola, Sean Callaghan, Gabriel Avillion, Xuduo Li, Nathan Beutler, Peter Yong, Fabio Anzanello, Thomas F. Rogers, Dennis R. Burton, Joseph G. Jardine, Ian A. Wilson, Raiees Andrabi

**Affiliations:** 1 Department of Immunology and Microbiology, The Scripps Research Institute, La Jolla, California, United States of America; 2 Consortium for HIV/AIDS Vaccine Development (CHAVD), The Scripps Research Institute, La Jolla, California, United States of America; 3 Department of Integrative Structural and Computational Biology, The Scripps Research Institute, La Jolla, California, United States of America; 4 Department of Medicine, Perelman School of Medicine, University of Pennsylvania, Philadelphia, Pennsylvania, United States of America; 5 Department of Microbiology, Perelman School of Medicine, University of Pennsylvania, Philadelphia, Pennsylvania, United States of America; 6 Division of Infectious Diseases, Department of Medicine, University of California, San Diego, La Jolla, California, United States of America; 7 IAVI Neutralizing Antibody Center, The Scripps Research Institute, La Jolla, California, United States of America; 8 Ragon Institute of Massachusetts General Hospital, Massachusetts Institute of Technology, and Harvard University, Cambridge, Massachusetts, United States of America; 9 Skaggs Institute for Chemical Biology, The Scripps Research Institute, La Jolla, California, United States of America; NIEHS: National Institute of Environmental Health Sciences, UNITED STATES OF AMERICA

## Abstract

Broadly neutralizing antibodies (bnAbs) targeting conserved regions of the betacoronavirus spike are important for pan-betacoronavirus protection and pandemic preparedness. Here, we report the isolation of a human monoclonal antibody, CC65.1, from a SARS-CoV-2 convalescent donor that targets the conserved S2 stem helix region. CC65.1 neutralizes various sarbecoviruses, including SARS-CoV-2, and binds to the MERS-CoV spike but lacks MERS-CoV-neutralizing activity due to insufficient binding affinity. We utilized directed evolution to enhance the binding affinity of CC65.1 for the MERS-CoV S2 stem helix, yielding engineered antibody variants with newly acquired MERS-CoV-neutralizing activity. High-resolution structural analysis reveals key paratope mutations that enhance binding and stabilize epitope engagement. Our findings demonstrate the potential of in vitro affinity maturation to expand the neutralization breadth of stem-helix-targeting antibodies across divergent betacoronaviruses. This work supports the development of engineered bnAbs for broadly protective betacoronavirus countermeasures and provides a strategy for achieving cross-lineage neutralization.

## Author summary

The persistent emergence of new SARS-CoV-2 variants of concern that evade neutralizing antibodies, together with other zoonotic betacoronaviruses with pandemic potential, has provided strong motivation to develop broadly neutralizing antibodies (bnAbs) that target conserved regions of the spike protein in sarbecoviruses and other betacoronaviruses. Here, we employed a directed evolution strategy to engineer the sarbecovirus-neutralizing antibody CC65.1, which targets the conserved S2 stem helix, to enhance its binding affinity for the MERS-CoV stem helix region, thereby conferring MERS-CoV neutralization. High-resolution structural studies of engineered CC65.1 revealed that key mutations reshape the paratope to better accommodate and stabilize the MERS-CoV S2 stem helix, resulting in increased binding affinity and neutralization potency. This study emphasizes the critical role of in vitro affinity maturation in expanding the neutralization breadth of stem helix antibodies and provides insights for antibody engineering against betacoronaviruses.

## Introduction

The ongoing evolution and emergence of pathogenic coronaviruses, including Severe Acute Respiratory Syndrome Coronavirus 2 (SARS-CoV-2) and Middle East Respiratory Syndrome Coronavirus (MERS-CoV), continue to pose a significant threat to global public health [1–3]. The COVID-19 pandemic has underscored the urgent need for broadly protective countermeasures against current and future zoonotic coronaviruses with pandemic potential [4,5]. Among the four coronavirus genera, betacoronaviruses harbor multiple high-risk pathogens, including SARS-CoV-1, SARS-CoV-2, and MERS-CoV, with the latter exhibiting a high case fatality rate [6–9]. However, the antigenic diversity among betacoronavirus spike glycoproteins, particularly within the immunodominant receptor-binding domain (RBD), presents a formidable challenge for eliciting broadly neutralizing antibodies (bnAbs) that can cross-protect against different betacoronavirus lineages [9–12]. The conserved S2 subunit of the spike protein, particularly the stem helix region, has emerged as a promising target for cross-reactive antibodies due to its structure and sequence conservation across betacoronavirus lineages [13–15]. Recent in situ cryo-ET studies of SARS-CoV-2 show that stem-helix epitopes are only partially exposed on the prefusion spike, and that the bnAbs targeting this site likely neutralize by engaging the transiently exposed stem-helix during viral entry and blocking HR2-mediated back-zippering, thereby arresting spike refolding and membrane fusion [16,17].

Here, we report the isolation and characterization of a human monoclonal antibody (CC65.1) targeting the S2 stem helix that neutralizes sarbecoviruses and exhibits cross-reactivity with the MERS-CoV spike, although it does not neutralize MERS-CoV. To overcome this limitation, we utilized directed evolution to enhance the binding affinity of CC65.1 for the MERS-CoV stem helix and generated engineered antibody variants that acquired MERS-CoV-neutralizing activity. High-resolution structural studies reveal the molecular basis of this expanded neutralization breadth and provide mechanistic insights into epitope engagement and structural adaptation. Overall, our work demonstrates that in vitro affinity maturation can be leveraged to broaden the neutralization breadth of existing antibodies toward divergent betacoronavirus lineages, including MERS-CoV, thereby supporting the development of broadly protective countermeasures as critical components of preparedness against future pandemic and endemic coronaviruses.

## Results

### Isolation and characterization of the S2 stem helix bnAb CC65.1 from a SARS-CoV-2 convalescent human donor

**Fig 1:**
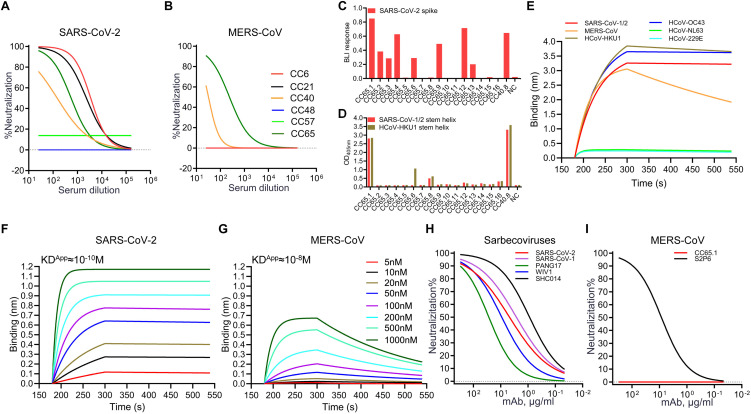
Identification of a stem-helix–targeting antibody with broad betacoronavirus binding and sarbecovirus neutralization activity. (**A-B)** Neutralization curves of sera from SARS-CoV-2 convalescent donors (CC6, CC21, CC40, CC48, CC57, and CC65) to SARS-CoV-2 **(A)** and MERS-CoV **(B)**. **(C)** BioLayer Interferometry (BLI) binding of 16 mAbs isolated from CC65 donor to SARS-CoV-2 S-2P protein. **(D)** Binding of 16 isolated mAbs from CC65 donor to SARS-CoV-1/2 and HCoV-HKU1 S2 stem helix peptides by ELISA. SARS-CoV-1/2 represents both SARS-CoV-1 and SARS-CoV-2 that have the same stem helix sequence. The binding is shown as absorbance at 405 nm (OD_405nm_). S2 stem helix mAb, CC40.8, was used as a positive control [20], and supernatant from mock-transfected Expi293 cells was used as a negative control. NC, negative control. **(E)** BLI binding curves of CC65.1 to S2 stem helix peptides from betacoronaviruses (SARS-CoV-1/2, MERS-CoV, HCoV-HKU1, and HCoV-OC43) and alphacoronaviruses (HCoV-NL63 and HCoV-229E). **(F-G)** BLI binding curves of CC65.1 to SARS-CoV-2 **(F)** and MERS-CoV **(G)** S-2P proteins. CC65.1 was captured on an AHC biosensor, followed by exposure to varying concentrations of S-2P protein. KD^App^ was determined using a 1:1 binding model with ForteBio Data Analysis software. (**H-I**) Neutralization curves of CC65.1 against pseudoviruses of ACE2-utilizing sarbecoviruses **(H)**, including clade 1a (SARS-CoV-1, WIV1, and SHC014) and clade 1b (SARS-CoV-2 and Pang17), and MERS-CoV **(I)**. The stem helix bnAb, S2P6, was used as a positive control [21].

To identify bnAbs targeting the conserved S2 stem helix of betacoronaviruses, we first screened sera from six SARS-CoV-2 convalescent donors (CC6, CC21, CC40, CC48, CC57, and CC65) for neutralizing activity against SARS-CoV-2 (sarbecovirus) and MERS-CoV (merbecovirus) pseudoviruses, which belong to different betacoronavirus lineages (Fig 1A-1B). Among these, only sera from CC40 and CC65 show cross-neutralization, with that from donor CC65 exhibiting notably stronger neutralizing activity against MERS-CoV. Based on this observation, we focused on CC65 for the isolation of potential stem helix bnAbs. Except for the stem helix region, sequence identity between the SARS-CoV-2 and HCoV-HKU1 (embecovirus) spikes is low. Thus, SARS-CoV-2 and HCoV-HKU1 recombinant soluble spike proteins with double proline substitutions (S-2P) were selected as baits to sort CD3^-^CD4^-^CD8^-^CD14^-^CD19 ⁺ CD20 ⁺ IgG ⁺ IgM^−^ SARS-CoV-2^+^HCoV-HKU1^+^ B cells from peripheral blood mononuclear cells (PBMCs) of donor CC65 (S1A Fig), thereby preferentially enriching for antibodies targeting conserved rather than lineage-specific epitopes. A total of 16 cross-reactive IgG ⁺ B cells were isolated, and their paired heavy and light chain sequences were recovered and expressed as recombinant monoclonal antibodies (mAbs). These mAbs were screened for binding to the SARS-CoV-2 S-2P protein and to S2 stem helix peptides derived from SARS-CoV-1/2 and HCoV-HKU1 (Fig 1C-1D). Eight of the 16 CC65 mAbs exhibit binding to the SARS-CoV-2 spike, and one (CC65.1) demonstrates the strongest stem helix binding activity (Fig 1D-1E).

Based on the conserved nature of the S2 stem helix within betacoronaviruses, we next evaluated the breadth of CC65.1. The antibody binds to multiple betacoronavirus spike proteins. For cell surface-expressed spikes, CC65.1 exhibits reduced binding from SARS-CoV-2 to SARS-CoV-1 and further to MERS-CoV (S1B Fig). For S-2P proteins, binding of CC65.1 to SARS-CoV-2 and SARS-CoV-1 is comparable and stronger than that to MERS-CoV (Figs 1F-1G and S1C). Consistent with its spike binding profile (Figs 1F-1G and S1B-S1E), CC65.1 also binds to S2 stem helix peptides from betacoronaviruses but not from the more phylogenetically distant alphacoronaviruses (HCoV-NL63 and HCoV-229E) (Fig 1E). We then assessed the neutralization breadth and potency of CC65.1 against a panel of sarbecoviruses, including clade 1a (SARS-CoV-1, WIV1, and SHC014) and clade 1b (SARS-CoV-2 and Pang17), as well as MERS-CoV. CC65.1 neutralizes all tested sarbecoviruses with half-maximal inhibitory concentrations (IC₅₀) ranging from 0.87 to 34.26 µg/mL but fails to neutralize MERS-CoV, even at the highest tested concentration of 300 µg/mL (Fig 1H-1I).

Given that neutralization potency often correlates with binding affinity [18,19], we hypothesized that CC65.1 may exhibit insufficient binding affinity for the MERS-CoV spike or stem helix region to achieve neutralization. To test this notion, we compared the binding affinity of CC65.1 to various betacoronavirus S-2P proteins and stem helix peptides. CC65.1 binds to SARS-CoV-1 and SARS-CoV-2 spikes and stem helix peptides with “apparent affinity” dissociation constants (KD^APP^) in the 10^-10^ M range (Figs 1F, S1C and S1 Table). In contrast, binding to the MERS-CoV spike (KD^APP^ ≈ 10^-8^ M) and stem helix peptide (KD^APP^ ≈ 10^-9^ M) is approximately 100-fold and 10-fold weaker, respectively (Fig 1E, 1G and S1 Table). Similar to other reported stem helix bnAbs [13], CC65.1 binds to the embecovirus HCoV-HKU1 and HCoV-OC43 S-2P proteins poorly (S1D-S1E Fig and S1 Table). Together, these results demonstrate that CC65.1 targets the conserved S2 stem helix region, exhibiting broad binding across betacoronaviruses. However, its reduced binding affinity for MERS-CoV likely accounts for its lack of neutralizing activity against this virus.

### Mapping and characterization of CC65.1 binding hotspots on the SARS-CoV-2 and MERS-CoV stem helix peptides

**Fig 2:**
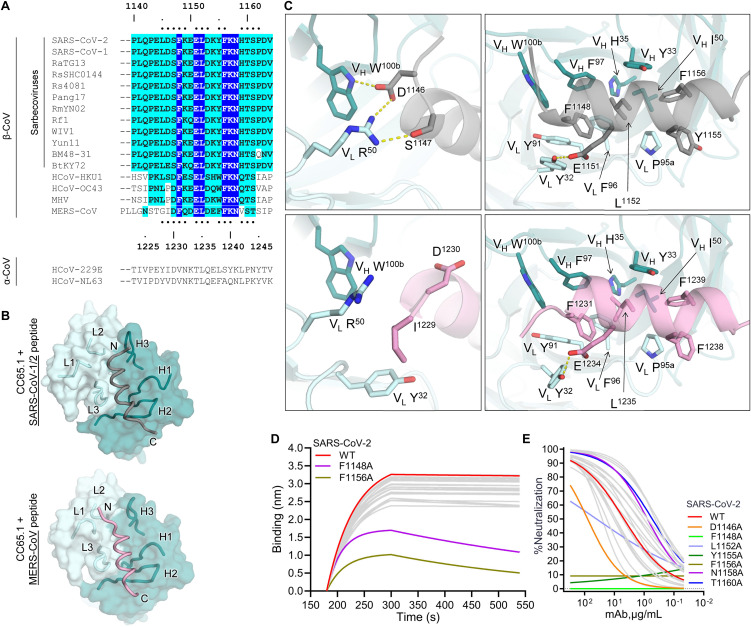
Structural and functional characterization of S2 stem helix recognition by CC65.1. **(A)** Sequence alignment between the S2 stem helix region of betacoronaviruses and alphacoronaviruses. SARS-CoV-2 and MERS-CoV numbering is shown on the top and bottom of the alignment, respectively. CC65.1 epitope residues (Buried surface area, BSA > 0 Å^2^) of SARS-CoV-1/2 and MERS-CoV are indicated by dots above and below the alignment, respectively. Conserved identical residues are highlighted in blue, while similar residues are in cyan, amino acids that score greater than or equal to 0 in the BLOSUM62 alignment score matrix are counted as similar here [23]. **(B)** Crystal structures of CC65.1 in complex with SARS-CoV-1/2 (grey) and MERS-CoV (pink) stem helix peptides. N- and C-terminus as well as CDR loops are indicated. Heavy and light chains of CC65.1 are shown in deep teal and pale cyan, respectively. **(C)** Details of molecular interactions between CC65.1 and stem helix peptides from SARS-CoV-1/2 (top) and MERS-CoV (bottom). S2 stem helix peptides of SARS-CoV-1/2 and MERS-CoV are shown in grey and pink, respectively. Hydrogen bonds and salt bridges are represented by yellow dashed lines. **(D)** BLI binding of CC65.1 to SARS-CoV-2 stem helix peptide alanine mutants spanning the whole peptide. The stem helix peptide mutants that most affect CC65.1 binding are shown in purple and olive in comparison to WT (red) and other stem helix mutants (grey). **(E)** Neutralization curves of CC65.1 to SARS-CoV-2 WT pseudovirus, pseudotyped variants with different individual stem helix alanine mutants, and N^1158^ glycan-knockout SARS-CoV-2 pseudovirus variants (N1158A and T1160A). The WT virus is shown in red, and virus mutants that substantially affect CC65.1 neutralization and glycan-knockout mutants are shown in assorted colors.

The S2 stem helix is conserved across betacoronaviruses, including SARS-CoV-2, SARS-CoV-1, and MERS-CoV (Fig 2A). To understand the mechanism by which CC65.1 neutralizes sarbecoviruses but not MERS-CoV, we determined crystal structures of CC65.1 Fab in complex with S2 stem helix peptides of SARS-CoV-1/2 and MERS-CoV at resolutions of 2.05 Å and 2.7 Å, respectively (Fig 2B and S2 Table). In both structures, the S2 stem helix peptides display a helical conformation, with all six CC65.1 complementarity-determining region (CDR) loops involved in peptide binding (Fig 2B). CC65.1 belongs to the public class of antibodies encoded by IGHV1–46/IGKV3–20, represented by S2P6 [21] (S2A-S2B Fig). This antibody class also includes CC68.109, CC99.103 [13], COV89–22, COV30–14, and COV93–03 [22]. Compared to CC40.8 [14,20], this class of antibodies binds the same S2 stem helix region, but with a different binding angle and orientation (S2B Fig). The C-terminal region of the S2 stem helix is highly conserved between SARS-CoV-1/2 (AA1148–1156) and MERS-CoV (AA1231–1239) (Fig 2A). Both stem helix peptides insert into a hydrophobic groove formed by the heavy and light chains of CC65.1 (Fig 2B). For example, F^1148^ of SARS-CoV-1/2 and F^1231^ of MERS-CoV extensively stack with aromatic residues of CC65.1 including V_H_ W^100b^, F^97^ and V_L_ Y^91^, Y^32^, and F^96^. SARS-CoV-1/2 L^1152^ and Y^1155^ (corresponding to L^1235^ and F^1238^ in MERS-CoV) also form hydrophobic interactions with V_H_ H^35^, Y^33^, I^50^ and V_L_ F^96^, P^95a^ of CC65.1. Moreover, the conserved E^1151^ (SARS-CoV-1/2)/E^1234^ (MERS-CoV) forms a hydrogen bond with V_L_ Y^32^ (Fig 2C). In contrast, the N-terminal region of the epitope is not conserved between SARS-CoV-1/2 and MERS-CoV (Fig 2A). Residues ^1146^DS^1147^ of SARS-CoV-1/2 form three hydrogen bonds and salt bridges with CC65.1 V_L_ R^50^ and V_H_ W^100b^, while the corresponding ^1229^ID^1230^ in MERS-CoV do not form these interactions; instead, I^1229^ forms hydrophobic interactions with V_L_ Y^32^ (Fig 2C). The loss of the polar interactions likely contributes to the reduced binding affinity of CC65.1 to MERS-CoV stem helix. Notably, V_L_ R^50^ is somatically mutated from its IGKV3–20 germline residue G^50^ that enables it to form these unique polar and charged interactions with SARS-CoV-1/2 (S2C Fig).

To verify the crucial residues for CC65.1 binding and neutralizing activity, we performed BLI binding and neutralization assays of CC65.1 to alanine scanning mutants of the SARS-CoV-2 stem helix peptide and pseudovirus, respectively. The F1148A and F1156A mutants substantially reduce the binding of CC65.1 to SARS-CoV-2 stem helix peptide (Figs 2D and S2D). Mutations F1148A, Y1155A and F1156A, as well as D1146A and L1152A, abolish or reduce neutralization by CC65.1 (Figs 2E and S2D). As SARS-CoV-2 has a glycosylation site at N^1158^, we generated N^1158^ glycan knockout mutants N1158A and T1160A to test the impact of glycosylation at N^1158^ on CC65.1 neutralization. Both mutations show ~10-fold increased neutralization potency, indicating that glycosylation at N^1158^ has some impact on CC65.1 neutralization.

We then compared structures of six mAbs targeting the SARS-CoV-2 S2 stem helix region, including four mAbs isolated from COVID-19 convalescent patients: CC65.1 (this study), CC40.8 [20], S2P6 (another mAb that belongs to the IGHV1–46/IGKV3–20 class) [21], and CV3–25 [24,25], as well as two mAbs, B6 and IgG22, isolated from spike-immunized mice [26,27] (S2A-S2B Fig). S2P6 adopts the same binding mode as CC65.1. Note that CC65.1 and S2P6 are both encoded by the same V genes, but with distinct HCDR3 loops (S2C Fig). Germline-encoded residues V_H_ Y^33^, H^35^, and V_L_ Y^32^, Y^91^ of both mAbs form a hydrophobic groove that interacts with the hydrophobic core of the S2 stem helix (S2C and S2E Figs). A V_H_ S56G somatic hypermutation of CC65.1 avoids a possible clash between the side chain of V_H_ S^56^ and the antigen. Other IGHV1–46/IGKV3–20 antibodies, including CC68.109, CC99.103 [13], and COV89–22 [22], share the same mutation, demonstrating a common and convergent mutation upon affinity maturation. On the other hand, V_H_ S^56^ of S2P6 is mutated to a histidine, stacking with F^1156^ (S2E Fig).

Compared to CC65.1, the epitope of CC40.8 extends more toward the N-terminus of the S2 stem helix (S2A Fig), and is recognized with a very different antibody binding mode (S2B Fig). Mouse antibodies B6 and Fab22 bind a more truncated epitope compared to CC65.1 that is translated along the groove between the heavy and light chains (S2A-S2B Fig). CV3–25 targets the S2 stem region with a completely different binding approach, and its epitope is more C-terminal compared to all the other mAbs analyzed here (S2A-S2B Fig). Finally, when modeled onto a SARS-CoV-2 spike trimer in the prefusion state (S2F Fig), all of these S2 stem-helix-targeting mAbs that bind the hydrophobic face of the stem helix would clash with the adjacent protomers of the prefusion spike trimer. Taken together, these structural and functional analyses define a conserved hydrophobic binding core that enables CC65.1 to broadly recognize the S2 stem helix across betacoronaviruses, while sequence divergence at the N-terminal likely weakens interactions of CC65.1 with MERS-CoV stem helix and limits its neutralization.

### Directed evolution engineering of CC65.1 enables MERS-CoV neutralization through enhanced binding affinity

**Fig 3:**
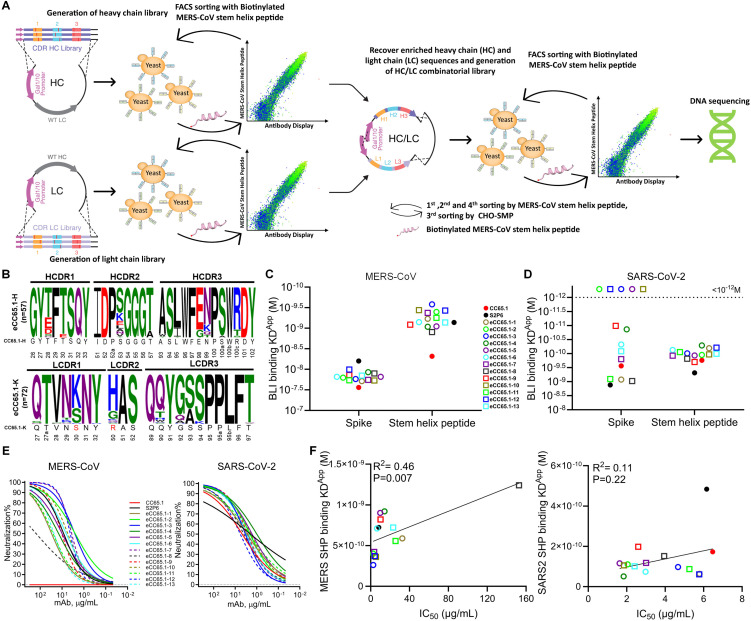
In vitro affinity maturation of CC65.1 by directed evolution conferring MERS-CoV neutralization. **(A)** Library strategy: the CC65.1 antibody library with single mutations at each CDR loop was displayed as molecular Fab on the surface of yeast cells. The CC65.1 heavy chain (HC) library with up to three mutations was paired with the original light chain (LC), while LC library was paired with the original HC. After four rounds of FACS sorting by MERS-CoV stem helix peptide and CHO-SMP, the HC library and LC library were amplified and combined into a combinatorial HC/LC library and further sorted for high binding clones by MERS-CoV stem helix peptide. Enriched clones with high binding affinity were sequenced, reformatted, and expressed as human IgG. **(B)** Identification of signature substitutions by comparison between CC65.1 and engineering CC65.1 (eCC65.1) variants using the program WebLogo. Clones from HC/LC library sort 4 were sequenced, sequences were aligned and sequence logos of CDRs of eCC65.1 HC and LC are shown. Sequences of CC65.1 CDRs are shown below each logo, the signature substitutions enrich among the eCC65.1 variants are highlighted in red. Sequence logos are indicated by colors representing their different biochemical properties: green for polar, blue for basic, red for acidic, black for hydrophobic and purple for N or Q residues. Kabat numbering is shown under the logos. **(C-D)** Comparison of the “apparent affinity” dissociation constants (KD^App^) of S2P6, parental CC65.1 and selected eCC65.1 mAbs against S-2P proteins and stem helix peptides from MERS-CoV **(C)** and SARS-CoV-2 **(D)**, different antibodies are shown in assorted shapes and colors. **(E)** Neutralization curves of parental CC65.1 and eCC65.1 mAbs against pseudotyped MERS-CoV (left) and SARS-CoV-2 (right). S2P6, a stem helix bnAb [21], was used as a positive control. **(F)** Plots showing correlation between KD^APP^ of S2P6, CC65.1 and selected eCC65.1 mAbs to MERS-CoV and SARS-CoV-2 stem helix peptides with neutralization potency (IC_50_) against their corresponding pseudoviruses. The same shapes and colors are employed to indicate different antibodies as in panel **C.** Correlations were determined by nonparametric Spearman correlation two-tailed test with 95% confidence interval. The Spearman correlation coefficient (R^2^) and p-values are indicated.

To further improve the neutralization potency to MERS-CoV as well as retain its broad reactivity across betacoronaviruses, CC65.1 was affinity-matured in vitro using the MERS-CoV stem helix peptide. We employed the Synthetic Antibody Maturation by multiple Point Loop library EnRichments (SAMPLER), a directed evolution strategy that rapidly enhances the antibody affinity by systematically introducing mutations in the CDRs [28–30]. Briefly, separate heavy chain (HC) and light chain (LC) libraries were created by introducing single mutations into each of the CDR loops and displayed on the surface of yeast as molecular Fab (Fig 3A). The HC library was paired with an unmodified LC, and vice versa. These libraries underwent four rounds of selection to enrich variants exhibiting high affinity and specificity for the MERS-CoV stem helix peptide. In rounds one, two, and four, cells were labeled with subsaturating concentrations of biotinylated MERS-CoV stem helix peptide, and the top 5–10% of peptide-binding cells, normalized for Fab surface display, were enriched. In the third round, cells were incubated with biotinylated, detergent-solubilized Chinese hamster ovary cell membrane proteins (CHO-SMP) to remove polyreactive clones (Fig 3A). Subsequently, enriched HC and LC variants were recombined into a combinatorial HC/LC library and subjected to an additional four rounds of selection to identify optimal HC/LC pairs that exhibited maximal binding affinity to the MERS-CoV stem helix peptide.

After the final selection round, compared with the cells from HC and LC sort 4, the enriched variants of HC/LC sort 4 can bind to the MERS-CoV stem helix peptide very strongly, even at very low peptide concentration (0.06 nM, HC/LC 92.4% vs HC 0.076% or LC 0.039%) (S3 Fig). These variants also show cross-reactivity with SARS-CoV-1/2, HCoV-HKU1, and HCoV-OC43 stem helix peptides (S4 Fig), confirming that the engineered CC65.1 (eCC65.1) mAbs retained broad binding activity. Plasmids encoding the HC and LC from more than 50 variants were amplified and sequenced. Sequence analysis of the engineered antibody variants from HC/LC sort 4 reveals mutations in CDRH1, CDRH2, and CDRH3 regions; however, none of these mutations shows strong enrichment (Fig 3B). In contrast, the light chain exhibits strong enrichment for two mutations: a serine-to-lysine substitution at position S30 in CDRL1 (S30K) and an arginine-to-histidine substitution at position R50 in CDRL2 (R50H) (Fig 3B). Compared with these two mutations, the enrichment of S93A in CDRL3 is slight.

A set of 13 eCC65.1 mAbs, named eCC65.1-1 through eCC65.1-13, were selected for further characterization (Figs 3C-3F, S5, S6A-S6C, S6E and S7). To ensure that directed evolution did not inadvertently increase polyreactivity, we performed polyspecificity reagent (PSR) ELISA assay and also assessed polyreactivity or autoreactivity in HEp2 cells. All of the selected eCC65.1 mAbs are negative in the PSR assays and a few of the selected eCC65.1 mAbs show some degree of polyreactivity or autoreactivity in HEp2 assay (S5 Fig). All of the affinity-matured antibodies bind the MERS-CoV stem helix peptide with higher affinity than the parental CC65.1 antibody, showing an average 9.1-fold increase in KD^APP^ values (range: 3.9-18.4-fold) (Figs 3C, S6C and S7D). This affinity improvement is primarily driven by a reduced dissociation rate (k_off_) (S6C and S7D Fig). Binding to the MERS-CoV S-2P protein also improves modestly (1.9-fold) compared to the parental CC65.1 (Figs 3C, S6C and S7B). Interestingly, most of the eCC65.1 variants exhibit a dramatic increase in binding affinity to the SARS-CoV-2 S-2P protein (average 109-fold) and a moderate enhancement in binding to the SARS-CoV-2 stem helix peptide (1.91-fold), despite these targets not being part of the directed evolution selection process (Figs 3D, S6C and S7A, S7C). The disparity in binding affinity improvement between MERS-CoV and SARS-CoV-2 S-2P proteins may reflect differences in epitope accessibility within the prefusion spike trimers of various betacoronaviruses.

To assess whether enhanced binding affinity translated into improved neutralization potency, IC_50_ values against MERS-CoV, SARS-CoV-2, and SARS-CoV-1 were determined (Figs 3E and S6C). eCC65.1 variants display similar neutralization potency against SARS-CoV-2 and SARS-CoV-1 with the parental antibody. All engineered variants, including eCC65.1-6 (V_L_ S30K and R50H), eCC65.1-12 (V_L_ S30K, R50H, and S93A) and eCC65.1-13 (V_L_ S30K, R50G, and S93A), which only have mutations in the light chain, show significantly improved MERS-CoV neutralization, with a median IC_50_ of 22.3 µg/mL (Figs 3E and S6A-S6C). eCC65.1-6 and eCC65.1-12 have higher MERS-CoV neutralization potency than eCC65.1-13, and eCC65.1-1, 4, 5, 8, 9, 11, and 13, which lack V_L_ S30K or R50H mutation, are not as potent as the others for MERS-CoV neutralization. Thus, these two mutations may have a greater effect on MERS-CoV neutralization potency than the other mutations. For naturally derived KV3-20 MERS-CoV-neutralizing human stem helix bnAbs, although basic amino acids (K and R) enriched on CDRL1 site 30 (7/22), V_L_ K^30^ (2/22) and H^50^ (0/22), seem to be rare among these antibodies, all eight KV3-20 MERS-CoV-non-neutralizing antibodies lack both V_L_ K^30^ and H^50^ (S6D Fig). In order to assess the role of these two mutations as well as S93A, which is enriched slightly in CDRL3, in improving MERS-CoV neutralization potency, eCC65.1-12 was selected for structural study.

The neutralization potency of eCC65.1 mAbs reveals a positive correlation with binding affinity to the MERS-CoV stem helix peptide, but not the MERS-CoV S-2P protein (Figs 3F and S6E). For SARS-CoV-2, binding affinity to both the stem helix peptide and S-2P protein show a positive correlation with neutralization potency, although the correlation with stem helix is not significant (Figs 3F and S6E). Despite a > 100-fold increase in apparent binding affinity to the SARS-CoV-2 S-2P protein for most of the eCC65.1 mAbs, such as eCC65.1-2, eCC65.1-5 and eCC65.1-10, no substantial improvement in SARS-CoV-2 stem helix binding activity and neutralization potency is observed (Figs 3E, S6C and S7A, S7C). This may be due to the parental CC65.1 already exhibiting sufficient spike and stem helix binding affinity, which ensures its neutralization (IC_50_ = 6.16 µg/mL) to SARS-CoV-2, and further binding affinity enhancement does not translate into improved neutralization potency beyond a certain threshold. In contrast, binding affinity improvements in eCC65.1 variants lead to a marked gain in neutralization capability to MERS-CoV. Together, these results demonstrate that using the SAMPLER platform for in vitro affinity maturation, we successfully engineered CC65.1 variants with enhanced binding affinity and newly acquired MERS-CoV-neutralizing activity. This highlights the potential of the in vitro affinity maturation strategy to expand antibody breadth and functionality against related, previously resistant viruses.

### Structural basis of MERS-CoV neutralization by eCC65.1

To further understand the mechanism by which enhanced binding affinity to the MERS-CoV stem helix peptide contributes to neutralization potency against MERS-CoV, we selected eCC65.1-12, which contains only three mutations in the light chain (V_L_ S30K, R50H, and S93A) and exhibits improved binding and neutralization activity against MERS-CoV, for detailed functional and structural characterization. eCC65.1-12 retains the broad reactivity of parental CC65.1, binding to stem helix peptides and cell surface-expressed spike proteins from multiple betacoronaviruses, but not alphacoronaviruses such as HCoV-NL63 and HCoV-229E (S8A-S8B Fig and S1 Table). Consistent with conservation of the stem helix in betacoronaviruses, the enhanced binding to MERS-CoV stem helix translates into increased affinity for other betacoronavirus stem helix peptides, including SARS-CoV-1/2 (35.4-fold), HCoV-HKU1 (6.7-fold) and HCoV-OC43 (7.6-fold) (Figs 1E, S8A and S1 Table), although the binding to S-2P proteins is not significantly improved except for SARS-CoV-2 (Figs 1F-1G, S1C-S1E, S8C-S8G and S1 Table). Notably, the binding of eCC65.1-12 to cell surface-expressed full-length HCoV-HKU1 and MERS-CoV spike is improved (S1B, S8B Figs and S1 Table). Both CC65.1 and eCC65.1-12 neutralize all tested sarbecoviruses (SARS-CoV-1, SARS-CoV-2, SHC014, PANG17, and WIV1), but only eCC65.1-12 neutralizes MERS-CoV with an IC_50_ of 4.18 µg/mL (S8H-S8I Fig). Despite weaker binding of both antibodies to SARS-CoV-1 spike compared to SARS-CoV-2 spike (Figs 1F, S1B-S1C and S8B-S8C, S8E), their neutralization potency against the two viruses remains comparable (Figs 1H, S6C and S8H–S8I).

**Fig 4:**
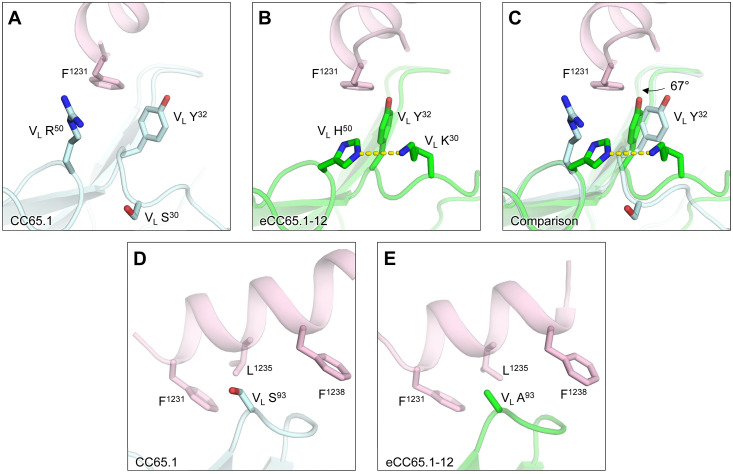
Structural basis of enhanced binding of eCC65.1-12 to the MERS-CoV S2 stem helix. Interactions of the MERS-CoV S2 stem helix peptide with CC65.1 and eCC65.1-12 are shown based on crystal structures. CC65.1 is shown in pale cyan and eCC65.1-12 in green, and the MERS-CoV stem helix peptide is shown in pink. Hydrogen bonds are represented by yellow dashed lines. The effects of mutations in eCC65.1-12 compared to WT at V_L_ residues 30 and 50 are shown in panels A-C, and that at V_L_ residue 93 is shown in panels D and **E.**

We further determined a crystal structure of eCC65.1-12 in complex with the MERS-CoV S2 stem helix peptide (Fig 4). Compared with parental CC65.1, eCC65.1-12 contains three mutations in the light chain, including S30K, R50H, and S93A. The R50H substitution induces a 67° rotation in the rotamer of the V_L_ Y^32^ side chain that enables V_L_ Y^32^ of eCC65.1-12 to form a T-shaped stacking interaction with MERS-CoV F^1231^^,^ which is highly conserved among betacoronaviruses (equivalent to SARS-CoV-2 F^1148^) (Fig 2A), likely strengthening the interaction (Fig 4A-4C). Indeed, in most of the eCC65.1 mAbs, V_L_ R^50^ is mutated, mainly to histidine (Figs 3B and S6B). Interestingly, eCC65.1-13 is almost identical to eCC65.1-12 except for one residue difference at V_L_ residue 50 (S6B Fig), where eCC65.1-13 contains a glycine instead of histidine. This one-residue difference leads to a ~ 6-fold decrease in neutralization potency of eCC65.1-13 against MERS-CoV (Figs 3E and S6C), further suggesting that the V_L_ H^50^ mutation enhances binding. V_L_ S30K forms a hydrogen bond with V_L_ H^50^ that may further stabilize the CDR conformations due to these new interactions between CDR L1 (K^30^, Y^32^) and L2 (H^50^) (Fig 4B-4C). V_L_ S93A also increases hydrophobic interactions with epitope residues F^1231^, L^1235^, and F^1238^ in MERS-CoV (Fig 4D-4E). Together, these findings demonstrate that in vitro affinity maturation of CC65.1 confers MERS-CoV neutralization while preserving its broad betacoronavirus reactivity. Structural analyses indicate that key light chain mutations reshape the paratope to strengthen interactions with conserved residues in the S2 stem helix, thereby enhancing binding affinity and enabling cross-lineage neutralization.

## Discussion

The emergence of pathogenic betacoronaviruses, such as SARS-CoV-1, SARS-CoV-2 and MERS-CoV, underscores the urgent need for bnAbs that can provide cross-lineage protection [31,32]. In this study, we show that sera from a SARS-CoV-2 convalescent donor (CC65) exhibit neutralizing activity against both SARS-CoV-2 and MERS-CoV, suggesting the presence of antibodies with dual neutralizing capacity. However, such antibodies were not isolated, likely due to the sorting strategy using SARS-CoV-2 and HCoV-HKU1 S-2P proteins as baits, rather than SARS-CoV-2 and MERS-CoV S-2P proteins as in our previous study [13]. Notwithstanding, a MERS-CoV stem-helix-binding antibody, CC65.1, was obtained. CC65.1 neutralizes SARS-CoV-2 and other ACE2-utilizing sarbecoviruses, but lacks MERS-CoV-neutralizing activity. Structural and biochemical analyses reveal that the lack of MERS-CoV neutralization is attributable to reduced binding affinity and fast dissociation rate (k_off_) of CC65.1 to MERS-CoV stem helix, which may be driven in part by sequence divergence at the N-terminal end of the S2 stem helix, thereby limiting its ability to effectively capture and retain the transiently exposed MERS-CoV stem helix to block formation of the postfusion hairpin and membrane fusion [16,17]. To overcome this, we employed a directed evolution strategy using the SAMPLER platform to enhance the binding affinity of CC65.1 to the MERS-CoV stem helix. The engineered variants, for example eCC65.1-12, acquire substantially enhanced binding affinity and reduced dissociation rate to MERS-CoV stem helix peptide, which may enable more efficient engagement and sustained occupancy of the transiently exposed MERS-CoV epitope, resulting in acquiring MERS-CoV-neutralizing activity while retaining broad reactivity to other betacoronaviruses.

High-resolution structural studies of eCC65.1-12 reveal the mechanistic basis for MERS-CoV neutralization potency improvement, highlighting key light chain mutations, such as S30K in CDRL1 and R50H in CDRL2, which reshape the paratope to better accommodate MERS-CoV S2 stem helix and stabilize the CDR conformations, rather than directly optimize the antigen-contacting residues of CC65.1. This non-intuitive structural solution would have been difficult to achieve by conventional rational design approaches that typically focus on direct interface optimization. Similar to this study, we previously analyzed how an in vitro affinity-matured mAb targeting the conserved CR3022 site of the SARS-CoV-2 RBD gained neutralization potency and breadth against SARS-CoV-2 variants while retaining SARS-CoV-1 neutralization [28]. These findings underscore how even subtle epitope differences within a conserved region can significantly affect neutralization and how in vitro affinity maturation by directed evolution can expand neutralization breadth.

Our study adds to the growing body of evidence supporting the S2 stem helix as a viable target for broad betacoronavirus countermeasures [21,33–35]. While the receptor-binding domain (RBD) remains the dominant target of most neutralizing antibodies, its high variability across betacoronavirus lineages limits its utility for pan-betacoronavirus countermeasures [36,37]. In contrast, the S2 stem helix is conserved in both structure and sequence and has critical function in virus entry, which makes it an ideal target for broadly protective betacoronavirus countermeasures [13,16,17,20,22,38]. However, some natural stem helix antibodies like CC65.1 may require binding affinity optimization to neutralize more antigenically distinct viruses such as MERS-CoV. This work demonstrates that in vitro affinity maturation guided by directed evolution can expand the breadth of existing bnAbs and enable cross-lineage neutralization, including against viruses not originally targeted by the immune response. Such approaches could be readily applied to optimize existing antibodies from virus-infected or vaccinated individuals to cover the full diversity of pathogenic and pre-emergent betacoronaviruses.

In conclusion, the successful engineering of CC65.1 into a cross-neutralizing antibody against both sarbecoviruses and MERS-CoV provides a blueprint for the development of next-generation betacoronavirus countermeasures. Future efforts should focus on combining broad S2-targeting antibodies with complementary RBD-targeting bnAbs or incorporating engineered stem helix immunogens into vaccine platforms [39]. These strategies will be vital for establishing broad and durable immunity against both current and future betacoronavirus threats, while advancing pandemic preparedness.

## Materials and methods

### Cell lines

FreeStyle293-F cells (Thermo Fisher Scientific Cat# R79007) were cultured in FreeStyle 293 Expression Medium (Gibco Cat# 12338018), and Expi293F cells (Gibco Cat# A14527) were maintained in Expi293 Expression Medium (Gibco Cat# A1435101). Both suspension cells were incubated in the shaker at 150 rpm, 37°C, 8% CO_2_. HEK293T cells, HeLa cells stably expressing hACE2 (HeLa-hACE2) and hDPP4 (HeLa-hDPP4) cells were grown in Dulbecco’s Modified Eagle Medium (DMEM) supplemented with 10% heat-inactivated FBS, 4mM L-Glutamine and 1% penicillin-streptomycin, in an incubator at 37°C, 5% CO_2_.

### Expression and purification of betacoronavirus recombinant soluble spike protein and antibody

To generate stable trimeric spike proteins [40,41], double proline (2P) substitutions were introduced into the S2 subunit at the following positions: K968/V969 in SARS-CoV-1, K986/V987 in SARS-CoV-2, V1060/L1061 in MERS-CoV, A1071/L1072 in HCoV-HKU1, and A1078/L1079 in HCoV-OC43. In addition, the furin cleavage sites of spikes (SARS-CoV-1: 664–667; SARS-CoV-2: 682–685; MERS-CoV: 748–751; HCoV-HKU1: 756–760 and HCoV-OC43: 762–766) were replaced with a “GSAS” linker. The trimerization domain of T4 fibritin (folden) was appended to the C-terminus, followed by an HRV-3C protease cleavage site, a 6 × His tag, and an AviTag, each separated by a GS linker, to facilitate purification and biotinylation. The recombinant soluble spike proteins with double proline substitutions (S-2P) proteins were expressed in FreeStyle293-F cells as previously described [42]. For purification, cell culture supernatants were collected and secreted spike proteins were purified with the HisPur Ni-NTA Resin (Thermo Fisher Scientific Cat# 88221) and eluted with 200 mM imidazole. The purified proteins were concentrated and further purified by size-exclusion chromatography by Superdex 200 Increase 10/300 GL column (GE Healthcare Cat# GE28990944) in PBS. Proteins were concentrated again and stored at -80°C for further use.

Monoclonal antibody (mAb) expression and purification were performed as previously reported [42]. In brief, the paired heavy and light chain were co-transfected into Expi293 cells using FectoPRO PolyPlus reagent (Polyplus Cat# 116–040). After 24h post-transfection, sodium valproic acid and glucose were added. After 4 days of incubation, cell supernatants were harvested, and mAbs were purified using Protein A and Protein G Sepharose (GE Healthcare Cat# 17061805).

### Flow cytometry B cell profiling and monoclonal antibody isolation

To isolate antigen-specific memory B cells, HCoV-HKU1 and SARS-CoV-2 S-2P proteins were used as probes for single cell sorting. B cell sorting and antibody isolation were performed as previously described [42]. The frozen PBMCs from donor CC65 were thawed and recovered in 10mL RPMI 1640 medium containing 50% FBS immediately before staining. The following reagents were used during staining: CD3 (APC Cy7, BD Pharmingen Cat# 557757), CD4 (APC-Cy7, Biolegend, Cat# 317418), CD8 (APC-Cy7, BD Pharmingen Cat# 557760), CD14 (APC-H7, BD Pharmingen Cat# 561384), CD19 (PerCP-Cy5.5, Biolegend Cat# 302230), CD20 (PerCP-Cy5.5, Biolegend Cat# 302326), IgG (BV786, BD Horizon Cat# 564230) and IgM (PE, Biolegend Cat# 314508). After staining by the above Ab mixture, cells were incubated with HCoV-HKU1 and SARS-CoV-2 S-2P proteins which were conjugated to streptavidin-AF488 (Thermo Fisher Scientific Cat# S11223) and streptavidin-AF647 (Thermo Fisher Scientific Cat# S21374), respectively, on ice for 30min. Prior to sorting, FVS510 Live/Dead stain (Thermo Fisher Scientific Cat# L34966) was added to exclude the dead cells. Cross-reactive spike specific B cells (SARS-CoV-2^+^HCoV-HKU1^+^CD19^+^CD20^+^CD3^-^CD4^-^CD8^-^CD14^-^IgM^-^IgG^+^) were sorted into 96-well plates. To amplify the variable regions of lgG heavy and light chain, RT-PCR and nested PCR were performed as previously described [13]. The purified DNA fragments were cloned into expression vectors encoding human IgG1, and Ig kappa/lambda constant domains, respectively, using HiFi DNA assembly (New England Biolabs Cat# E2621L).

### ELISA

The binding of 16 isolated mAbs from CC65 donor with SARS-CoV-1/2 and HCoV-HKU1 S2 stem helix peptides was assessed by ELISA. The 96-well half-area microplates were coated with 100 ng/well streptavidin (Jackson Immuno Research Labs Cat# 016-000-084) at 4°C overnight. The plates were washed three times with PBST (PBS + 0.05% Tween20) and blocked with 3% bovine serum albumin (BSA) in PBS for 2h at room temperature (RT). After removing the blocking buffer, plates were treated with biotinylated S2 stem helix peptides (5 μg/mL in 50 μL 1% BSA) for 1h at RT. Following additional washes, diluted antibodies (10 μg/mL in 50 μL 1% BSA) were added and incubated for 1h. Following washes, secondary antibody (Jackson ImmunoResearch Laboratories Cat# 109-055-008) was added in 1:1000 dilution for an additional 1h. After final washes, alkaline phosphatase substrate (Sigma-Aldrich Cat# S0942-200TAB) was added. Absorbance at 405 nm was measured after 30 min using VersaMax microplate reader (Molecular Devices).

### Antibody library generation

The CC65.1 heavy chain (HC) and light chain (LC) Fab libraries were generated as reported previously [28–30]. In brief, oligopools, which contained a single mutation in each complementarity-determining region (CDR), were synthesized (Integrated DNA Technologies). Then, the CDR1/2/3 mini-libraries were assembled into combinatorial heavy chain and light chain libraries. The pYDSI2w vector containing the bidirectional Gal1–10 promoter was used to display the Fab libraries on the yeast surface. V5 and c-Myc epitope tag at the C-terminal of HC and LC, respectively, were taken to measure the amount of Fab displayed on the yeast surface. The HC library was generated by cloning the HC CDR1/2/3 library into pYDSI2w vector, which already had the parental CC65.1 LC. The LC library was generated by cloning the LC CDR1/2/3 library into pYDSI2w vector, which already had the parental CC65.1 HC. In order to generate CC65.1 HC/LC combinational library, the HC and LC sequences were amplified from the sorted HC and LC libraries with primers overlapping in the Gal1–10 promoter. Then, the purified HC and LC fragments were ligated by HiFi DNA assembly (New England Biolabs Cat# E2621L) and amplified to get the LC–Gal1–10–HC product which could be inserted into the empty pYDSI2w vector to generate CC65.1 HC/LC library.

### Yeast transformation

The *Saccharomyces cerevisiae* YVH10 electrocompetent cells were mixed with 1 μg linearized pYDSI2w vector and 5 μg HC, LC or HC/LC DNA, then were transferred into a 0.2 cm electroporation cuvette (Bio-Rad Cat# 1652086) and inserted into a Gene Pulser Xcell Electroporation System (Bio-Rad Cat# 1652666) using the following settings: square wave, voltage = 500 V, pulse length = 15.0 ms, number of pulses = 1, pulse interval = 0, and cuvette = 2 mm. After electroporation, the yeast cells were moved into 25 mL YPD medium and cultured at 30°C for 1 hour with shaking at 200 rpm. Then, 2.5 μL of the yeast cells was diluted serially to estimate transformation efficiency with the colony-forming unit assay on synthetic complete agar plates without tryptophan (SC-Trp) (Sunrise Science Cat# 1710–300). The other cells were transferred to 250 mL SC-Trp medium (Sunrise Science Cat# 1709–500) with 1% penicillin/streptomycin (Corning Cat# 15323671) and shaken overnight at 30°C for further sorting.

### Yeast library labeling and sorting

After yeast transformation, the cells were passaged 1:20 next day, then induced at OD = 1.0 overnight in SGCAA medium [43]. For each library, 5 x 10^7^ cells were stained in the first round of sorting, 1 × 10^7^ cells in the following other 3 rounds. After being spun down and washed by PBSA (PBS with 1% BSA), the cells were incubated with biotinylated stem helix peptides or detergent-solubilized Chinese hamster ovary cell membrane proteins (CHO-SMP) at several non-depleting concentrations respectively for 30 min at 4°C. After washing by PBSA, the yeast cells were stained by anti-c-Myc antibody (FITC, Immunology Consultants Laboratory Cat# CMYC-45F), anti-V5 antibody (AF405, made in house), and streptavidin-APC (Invitrogen Cat# CSA1005) in 1:100 dilution for 20 min at 4°C. After final washing, the cells were resuspended in 1 mL PBSA and loaded on BD FACSMelody cell sorter, and top 5–10% of cells with high binding activity to a certain stem helix peptide concentration were sorted. Sorted cells were cultured in 2 mL SC-Trp medium (Sunrise Science Cat# 1709–500) supplemented with 1% Penicillin/Streptomycin (Corning Cat# 15323671) at 30°C overnight for further use.

### Yeast colony and DNA sequencing

After culturing the cells from HC/LC sort4 in SC-Trp medium (Sunrise Science Cat# 1709–500) overnight, the cells were diluted serially and grown on SC-Trp plates at 30°C overnight, then single colonies were picked and grown in SC-Trp medium at 30°C overnight. The DNA was extracted from yeast cells as reported previously [28–30], and the heavy chain and light chain regions were amplified from the DNA and Sanger sequenced. Bioedit (https://thalljiscience.github.io/) and WebLogo (https://weblogo.berkeley.edu/) were used to analyze the sequences.

### Pseudovirus production and neutralization assay

Sarbecovirus and MERS-CoV pseudoviruses were generated in HEK293T cells. Briefly, 12.5 μg pCMV-dR8.2 dvpr (Addgene Cat# 8455), 10 μg pBOB-Luciferase (Addgene Cat# 170674), and 2.5 μg sarbecovirus or MERS spike plasmid were mixed with transfection reagent Lipofectamine 2000 (ThermoFisher Scientific Cat# 11668019) and incubated for 15 min at RT and then transferred into HEK 293T cells. After 12-16h, the medium was changed with fresh complete medium (10% FBS, 4 mM L-Glutamine, and 1% Penicillin/Streptomycin). Supernatants containing pseudovirus were harvested after 48h post transfection, then aliquoted and frozen at -80 °C for further use.

Neutralization assay for ACE2-utilizing sarbecoviruses including clade 1a (SARS-CoV-1, WIV1, SHC014) and clade 1b (SARS-CoV-2 and Pang17) was performed by HeLa-hACE2 cell, MERS-CoV neutralization was performed using HeLa-hDPP4 cells. The 3-fold serially diluted antibodies were incubated with the same volume (25 μL/well) of pseudovirus for 1h at 37°C. After incubation, 50 μL of HeLa-hACE2 or HeLa-hDPP4 cells (10,000 cells/well) were added to each well. After 48h of incubation, luciferase activity was measured by BrightGlo substrate (Promega Cat# E2620) according to the manufacturer’s instructions. Fifty percent maximal inhibitory concentrations (IC_50_s) were determined by the dose-response-inhibition model with 5-parameter Hill slope equation in GraphPad Prism 7 (GraphPad Software).

### HEp2 epithelial cell polyreactive assay

Polyreactivity of engineered CC65.1 antibodies to human epithelial type 2 (HEp2) was determined by indirect immunofluorescence using HEp2 slides (Hemagen Cat# 902360). Briefly, mAb was diluted to 50 μg/mL in PBS and added onto immobilized HEp2 slides, followed by incubation for 30 min at RT. Slides were washed three times with PBS, and one drop of FITC-conjugated goat anti-human IgG was added onto each well and incubated for 30 min in the dark at RT. After washing, the coverslip was added to HEp2 slide with glycerol and the images were photographed on a Nikon fluorescence microscope to detect FITC signal.

### Polyspecificity reagent (PSR) ELISA

CHO-SMP, human insulin (Sigma-Aldrich Cat# I2643), single-stranded DNA (Sigma-Aldrich Cat# D8899) and double-stranded DNA (Sigma-Aldrich Cat# D8515) were coated onto 96-well half-area high-binding plates (Corning Cat# 3690) at 5 μg/mL in PBS and incubated overnight at 4°C. After washing with PBST, plates were blocked with 3% BSA for 2h at 37°C. The 5-fold serially diluted antibody starting from 50 μg/mL was added in plates to incubate for 1h at RT. The assay was performed as described in section “ELISA.”

### CELISA binding

Binding of CC65.1 and eCC65.1-12 to cell surface expressed spikes from different coronaviruses was evaluated by cell-based ELISA (CELISA) as described previously [44]. A total of 4x10^6^ HEK293T cells were seeded into each 10 cm culture dish and incubated at 37°C. After 24h, HEK293T cells were transfected with plasmids encoding full-length coronavirus spikes. After incubation at 37°C for another 48 hours, the cells were harvested and distributed into each well of 96-well round-bottom tissue culture plates (Corning Cat# 3799) for individual staining. Before staining, cells were washed three times with 200 µL FACS buffer (1xPBS, 2%FBS, 1mM EDTA), then were stained for 1h on ice with CC65.1 or eCC65.1-12 at 10 μg/mL in 50 µL staining buffer. After washing another three times with FACS buffer, the cells in each well were stained with 50 µL FACS buffer containing anti-human IgG Fc antibody (PE, diluted at 1:200, Southern Biotech Cat# 904009) and Zombie-NIR viability dye (diluted at 1:1000, BioLegend Cat# 423105) on ice in dark for 45min. Following the final washes with FACS buffer, the cells were resuspended and analyzed by flow cytometry (BD Lyrics cytometer), and the binding data were generated by calculating the Mean Fluorescence Intensity (MFI) using FlowJo 10 software. Mock-transfected 293T served as a negative control.

### BioLayer Interferometry binding (BLI)

The binding of parental Ab CC65.1 and engineered CC65.1 with betacoronavirus S-2P proteins and stem helix peptides was determined by BLI using the Octet RED96e system. For binding to S-2P protein, antibody was captured by anti-human IgG Fc capture (AHC) biosensors (ForteBio Cat# 185063) for 60s, followed by 60s baseline step with Octet buffer to remove unbound antibody. The sensors were then immersed into the S-2P protein in Octet buffer (PBS with 0.1% Tween) for 120s for association, followed by transferring into Octet buffer for 240s for dissociation. For stem helix peptide binding, N-terminal biotinylated stem helix peptides were diluted in Octet buffer and captured by the streptavidin (SA) biosensors (ForteBio Cat# 185020) for 60s, then the sensors were transferred into Octet buffer for 60s to remove the unbound peptides, followed by associating with monoclonal antibodies for 120s and dissociating in Octet buffer for 240s. The data were analyzed using the ForteBio Data Analysis software for correction, and the kinetic curves were fit to a 1:1 binding model. Different from intrinsic monovalent binding affinity of Fab, the IgG:spike or peptide binding can be a mixed population of 2:1 and 1:1 due to the multivalent nature of the IgG antibodies. Therefore, the term “apparent affinity” dissociation constants (KD^App^) are used to reflect the binding affinity between IgG antibodies and spike trimers or peptides tested.

### Expression and purification of Fab and single-chain variable fragment (scFv)

CC65.1 and eCC65.1-12 Fab plasmids were generated by introducing the stop codon right after the amino acid “KSC” in the heavy chain constant region. The truncated heavy chains were co-transfected with the corresponding light chains in Expi293F cells, and the supernatants were harvested 4 days post transfection. Fabs were purified with CaptureSelect CH1‑XL Affinity Matrix (Thermo Fisher Scientific Cat# 1943462250) and Superdex 200 Increase 10/300 GL column (GE Healthcare Cat# GE28-9909-44). The CC65.1 scFv genes were cloned with a C-terminal His_6_-tag in the heavy chain and light chain variable region (VH-VL) orientation, which linked together by a (G4S)_3_ to form a VH-VL-(G4S)_3_-His_6_ format. The production and purification methods of scFv with His tag are very similar with those in “Expression and purification of betacoronavirus recombinant soluble spike protein and antibody” section.

### Crystallization and X-ray structure determination

A mixture of antibodies and 10 × (molar ratio) stem helix peptides were screened for crystallization using the 384 conditions of the JCSG Core Suite (Qiagen) on our robotic CrystalMation system (Rigaku) at Scripps Research. Crystallization trials were set-up using the vapor diffusion method in sitting drops containing 0.1 μL of protein and 0.1 μL of reservoir solution. Diffraction-quality crystals were obtained in the following conditions:

CC65.1 scFv/ SARS-CoV-2 S2 stem peptide (11 mg/mL): 20% (w/v) PEG3350 and 0.2 M CaCl_2_ at 20°C; CC65.1 Fab/ MERS-CoV S2 stem peptide (15 mg/mL): 2% (v/v) PEG400, 2 M ammonium sulfate, and 0.1 M HEPES pH 7.5 at 20°C; eCC65.1-12 Fab/ MERS-CoV S2 stem peptide (16 mg/mL): 0.2 M ammonium sulfate, 25% (w/v) PEG 4000, 0.1 M sodium acetate pH 4.6 at 20°C

All crystals appeared on day 7 and were harvested on day 10. Before flash cooling in liquid nitrogen for X-ray diffraction studies, crystals were equilibrated in reservoir solution supplemented the following cryoprotectants:

CC65.1 scFv/ SARS-CoV-2 S2 stem peptide: 20% ethylene glycol; CC65.1 Fab/ MERS-CoV S2 stem peptide: 20% ethylene glycol; eCC65.1-12 Fab/ MERS-CoV S2 stem peptide: 10% ethylene glycol.

Diffraction data were collected at cryogenic temperature (100 K) at the Advanced Light Source on beamline 5.0.1, Stanford Synchrotron Radiation Lightsource (SSRL) on Scripps/Stanford beamline 12–1, and Advanced Photon Source at the Argonne National Laboratory on beamline 23-ID-B. Data were processed with HKL2000 [45] (S2 Table). Structures were solved by molecular replacement (MR) using PHASER [46] with PDB 7SJS as the MR model. Iterative model building and refinement were carried out in COOT and PHENIX [47,48], respectively (S2 Table). Epitope and paratope residues, as well as their interactions, were identified by accessing PISA at the European Bioinformatics Institute (http://www.ebi.ac.uk/pdbe/prot_int/pistart.html) [49].

### Statistical analysis

Statistical analysis was performed using Graph Pad Prism 7, USA. IC_50_ titers were compared using the non-parametric unpaired Mann-Whitney-U test. Correlations were determined by nonparametric Spearman correlation two-tailed test with 95% confidence interval. The Spearman correlation coefficient (R^2^) and p-value are indicated. Groups of data were compared using the Kruskal-Wallis non-parametric test. Dunnett’s multiple comparison test was also performed between experimental groups. Data were considered statistically significant when p < 0.05.

## Supporting information

S1 Fig B cell sorting strategy and binding ability of parental CC65.1. (A) Gating strategy of single cross-reactive lgG^+^ B cell sorting from CC65 donor. The CD19^+^CD20^+^CD3^−^CD4^−^CD8^−^CD14^−^IgM^−^IgG^+^HCoV-HKU1^+^SARS-CoV-2^+^ cells were sorted. (B) Binding of CC65.1 to the cell surface-expressed spikes from different alphacoronaviruses and betacoronaviruses. Binding was tested by CELISA, and mean fluorescence intensity (MFI) shown. (C-E) BLI binding curves of CC65.1 with SARS-CoV-1 (C), HCoV-HKU1 (D), and HCoV-OC43 (E) S-2P proteins. The “apparent affinity” dissociation constants (KD^APP^) is indicated.
(PDF)

S2 Fig Comparison of S2 stem-helix-targeting antibodies. (A) Epitopes of different stem-helix-targeting antibodies. Epitope residues (BSA > 0 Å^2^) of each antibody are indicated by dots under the sequence. Peptides used for each crystallization study are indicated as gray boxes. (B) Structures of CC65.1, CC40.8, S2P6, B6, CV3–25, and Fab22 in complex with SARS-CoV-1/2 S2 stem helix peptide. The SARS-CoV-1/2 S2 stem helices are represented by green tubes. N- and C-terminus of the bound antigens are indicated in the figure. (C) Sequence alignment between CC65.1, S2P6, and their putative germline sequences IGHV1–46*01 and IGKV3–20*01. Paratope residues shown in Fig S2E are highlighted in yellow. Residues conserved in all aligned sequences are labelled by an asterisk (*), whereas a colon (:) and a period (.) indicate strongly similar and weakly similar sequences, respectively as calculated by Clustal Omega [50]. Kabat numbering is shown under the sequence alignment. (D) Epitope mapping of CC65.1 by alanine scanning mutants of the SARS-CoV-2 stem helix peptide and pseudovirus using BLI and neutralization assay. The IC_50_ fold change (n-fold) was calculated by dividing the mutant value by the WT value. For IC50, n-fold >5 is indicated as cyan. The binding response value whose % change (compared to WT peptide) is < 70% are indicated in purple. N.A., not available. (E) Structural comparisons of conserved (left) and non-conserved (right) interactions with the SARS-CoV-1/2 stem helix between the two IGHV1–46*01/IGKV3–20*01 antibodies CC65.1 and S2P6. (F) Structural superimpositions of stem helix antibody crystal structures onto the SARS-CoV-2 spike prefusion structure. The structures of CC65.1 (teal), CC40.8 (yellow), S2P6 (purple), B6 (grey), CV3–25 (brown), and Fab22 (blue) in complex with SARS-CoV-2 S2 stem helix peptides were superimposed onto the SARS-CoV-2 spike prefusion structure (PDB 6XR8). All antibodies clash with the other protomers of the spike protein in prefusion state. Structures of CC40.8, S2P6, B6, CV3–25 and Fab22 are from PDBs 7SJS, 7RNJ, 7M53, 7NAB, and 7S3N, respectively.
(PDF)

S3 Fig Representative FACS plots of CC65.1 HC, LC and HC/LC libraries in the 4^th^ sorting. Surface Fab display frequency was determined by staining with AF405-anti-V5 antibody (HC) and FITC-anti-c-Myc antibody (LC). Cells were also labeled with different subsaturating concentrations of biotinylated MERS-CoV stem helix peptide, or without antigen. Labeled cells were further stained with APC-conjugated streptavidin. FACS analysis was performed by FlowJo. SHP, stem helix peptide.
(PDF)

S4 Fig Binding of yeast cells from the 4^th^ sort of HC/LC library to stem helix peptides of SARS-CoV-1/2, HCoV-HKU1, and HCoV-OC43. AF405-anti-V5 antibody (HC) and FITC-anti-c-Myc antibody (LC) were used in combination to determine the cell surface Fab display frequency. Cells were also labeled with different biotinylated betacoronavirus stem helix peptides at a subsaturating concentration (0.06 nM), or without antigen. Labeled cells were further stained with APC-conjugated streptavidin. FACS analysis was performed by FlowJo.
(PDF)

S5 Fig Evaluation of selected eCC65.1 mAbs for polyreactivity. (A) PSR ELISA for binding to CHO-SMP, insulin, single-stranded DNA (ssDNA) and double-stranded DNA (dsDNA). (B) Binding to immobilized HEp2 epithelial cells. Bococizumab, which was used as a positive control, is a humanized mAb targeting the low-density lipoprotein (LDL) receptor-binding domain of PCSK9, and it has been studied in phase I–III clinical trials [51]. Den3 was used as a negative control. Positive and negative controls for the HEp2 kit assay were provided by the manufacturer.
(PDF)

S6 Fig Sequence analysis, binding affinity, and neutralizing potency of selected eCC65.1 mAbs. Sequence alignment of the heavy chain (A) and light chain (B) variable regions of CC65.1, selected eCC65.1 mAbs, and their putative germline sequences IGHV1-46*01 and IGKV3-20*01. CDRs are highlighted in different colors, yellow for HCDR1 and LCDR1, red for HCDR2 and LCDR2, and cyan for HCDR3 and LCDR3. Kabat numbering is shown above the sequence alignment. (C) Summary table of binding affinity and neutralizing potency of S2P6, parental CC65.1 and selected eCC65.1 mAbs. IC_50_ values of S2P6, CC65.1 and eCC65.1 mAbs are shown in the upper table. Binding affinity of these antibodies to S-2P proteins and stem helix peptides of SARS-CoV-2 and MERS-CoV was tested by BLI, response values, KD^APP^, kon and koff were analyzed by fitting to a 1:1 binding model. (D) Identification of signature residues on LCDRs between naturally occurring KV3-20-derived MERS-CoV-neutralizing (top, n = 22) and MERS-CoV-non-neutralizing (bottom, n = 8) human stem helix mAbs. Sequences were aligned and sequence logos of LCDRs are shown. Sequences of CC65.1 LCDRs are shown between the logos, the signature substitutions enriched among the eCC65.1 antibodies are highlighted in red. Kabat numbering is shown under the logos. KV3-20-derived MERS-CoV-neutralizing human stem helix bnAbs include S2P6 [21], CC9.104, CC9.106, CC9.111, CC9.113, CC9.130, CC9.131, CC24.107, CC25.101, CC25.104, CC68.104, CC68.109, CC92.133, CC92.147, CC99.103, CC99.104, CC99.105 [13], COV89-22, COV30-14, COV72-37, COV44-26 and COV44-74 [22]. KV3-20-derived MERS-CoV-non-neutralizing human stem helix mAbs contain CC65.1, CC24.105, CC25.108, CC25.112, CC67.105, CC67.130, CC95.102 [13] and COV93-03 [22]. Sequence logos are indicated by colors representing their different biochemical properties: green for polar, blue for basic, red for acidic, black for hydrophobic and purple for N or Q residues. (E) Correlation analysis between KD^APP^ values of S2P6, CC65.1 and selected eCC65.1 mAbs for MERS-CoV and SARS-CoV-2 S-2P proteins and neutralization potency (IC_50_) against their corresponding pseudoviruses. Different antibodies are shown in assorted shapes and colors. Correlations were determined by nonparametric Spearman correlation two-tailed test with 95% confidence interval. The Spearman correlation coefficient (R^2^) and p-values are indicated.
(PDF)

S7 Fig BLI binding curves of S2P6, parental CC65.1 and selected eCC65.1 mAbs to spike proteins and stem helix peptides of SARS-CoV-2 and MERS-CoV. All BLI binding curves related to the binding data of antibodies to S-2P proteins (A-B) and stem helix peptides (C-D) of SARS-CoV-2 and MERS-CoV shown in Fig S6C are displayed here. The kinetic curves were fit to a 1:1 binding model. S2P6 was used as a positive control. Different antibodies are shown in assorted lines and colors.
(PDF)

S8 Fig Neutralizing potency and binding ability of eCC65.1-12 to betacoronaviruses. (A) BLI binding of eCC65.1-12–25-mer stem helix peptides derived from different coronavirus spikes. (B) Binding of eCC65.1-12 to betacoronavirus and alphacoronavirus spikes expressed on the HEK293T cell surface. MFI, Mean Fluorescence Intensity. (C-G) BLI binding curves of eCC65.1-12 to all five human betacoronavirus S-2P proteins. eCC65.1-12 was captured on an AHC biosensor, followed by exposure to varying concentrations of S-2P protein. The KD^App^ was determined using a 1:1 binding model with ForteBio Data Analysis software. (H-I) Neutralization potency of eCC65.1-12 to sarbecoviruses and MERS-CoV, with S2P6 as a positive control.
(PDF)

S1 Table Summary table of mean fluorescence intensity (MFI) and binding affinity of parental CC65.1 and eCC65.1-12 mAbs. MFI values of CC65.1 and eCC65.1-12 to betacoronavirus and alphacoronavirus spikes expressed on the HEK293T cell surface at different mAb concentrations are shown. Mock-transfected HEK293T cells were used as a negative control, and MFI was calculated by FlowJo. Binding affinity of these antibodies to S-2P proteins and stem helix peptides of betacoronaviruses was tested by BLI. KD^APP^, kon and koff were analyzed by fitting to a 1:1 binding model. N.D., not detected.
(PDF)

S2 Table X-ray data collection and refinement statistics. (PDF)
