# Correction: Directed evolution of a stem-helix–targeting antibody enables MERS-CoV cross-neutralization through enhanced binding affinity

**DOI:** 10.1371/journal.ppat.1014636

**Published:** 2026-09-28

**Authors:** The PLOS Pathogens Staff

The references 18 through 23 were out of order and reference 52 was omitted. Please see the corrected order for references 18 through 23 and reference 52 below.

18. Parren PW, Burton DR. The antiviral activity of antibodies in vitro and in vivo. Adv Immunol. 2001;77:195–262. https://doi.org/10.1016/s0065-2776(01)77018-6 PMID: 11293117
19. Rappazzo CG, Tse LV, Kaku CI, Wrapp D, Sakharkar M, Huang D, et al. Broad and potent activity against SARS-like viruses by an engineered human monoclonal antibody. Science. 2021;371(6531):823–9. https://doi.org/10.1126/science.abf4830 PMID: 33495307
20. Zhou P, Yuan M, Song G, Beutler N, Shaabani N, Huang D, et al. A human antibody reveals a conserved site on beta-coronavirus spike proteins and confers protection against SARS-CoV-2 infection. Sci Transl Med. 2022;14(637):eabi9215. https://doi.org/10.1126/scitranslmed.abi9215 PMID:35133175
21. Pinto D, Sauer MM, Czudnochowski N, Low JS, Tortorici MA, Housley MP, et al. Broad betacoronavirus neutralization by a stem helix-specific human antibody. Science. 2021;373(6559):1109–16. https://doi.org/10.1126/science.abj3321 PMID: 34344823
22. Dacon C, Peng L, Lin T-H, Tucker C, Lee C-CD, Cong Y, et al. Rare, convergent antibodies targeting the stem helix broadly neutralize diverse betacoronaviruses. Cell Host Microbe. 2023;31(1):97–111.e12. https://doi.org/10.1016/j.chom.2022.10.010 PMID: 36347257
23. Henikoff S, Henikoff JG. Amino acid substitution matrices from protein blocks. Proc Natl Acad Sci U S A. 1992;89(22):10915–9. https://doi.org/10.1073/pnas.89.22.10915 PMID: 1438297
52. Williams CJ, Headd JJ, Moriarty NW, Prisant MG, Videau LL, Deis LN, et al. MolProbity: More and better reference data for improved all-atom structure validation. Protein Sci. 2018;27(1):293–315. Epub 20171127. https://doi.org/10.1002/pro.3330 PMID: 29067766

The publisher apologizes for the error.
