# Supplementary material for: Directed evolution of a stem-helix–targeting antibody enables MERS-CoV cross-neutralization through enhanced binding affinity
Source: PLoS Pathog. 2026 Aug 7;22(8):e1014393. doi: 10.1371/journal.ppat.1014393 (PMC13480645; doi:10.1371/journal.ppat.1014393)
Supplement: Supplementary file 1 [file ppat.1014393.s001.pdf]

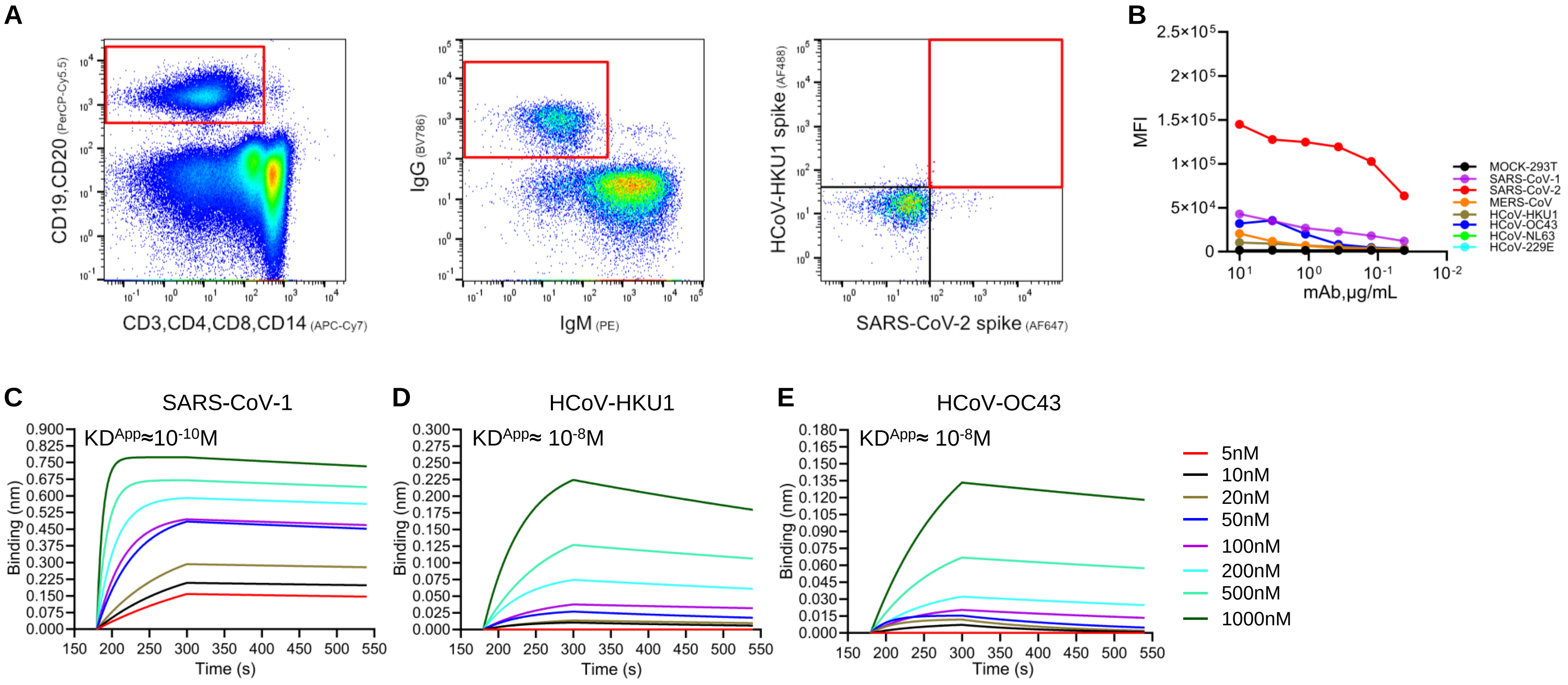
A
CD19,CD20 (PerCP-Cy5.5)
CD3,CD4,CD8,CD14 (APC-Cy7)
IgG (BV786)
IgM (PE)
HCoV-HKU1 spike (AF488)
SARS-CoV-2 spike (AF647)
B
MFI
mAb,μg/mL
MOCK-293T
SARS-CoV-1
SARS-CoV-2
MERS-CoV
HCoV-HKU1
HCoV-OC43
HCoV-NL63
HCoV-229E
C
SARS-CoV-1
KDApp≈10-10M
D
HCoV-HKU1
KDApp≈ 10-8M
E
HCoV-OC43
KDApp≈ 10-8M
Binding (nm)
Time (s)
5nM
10nM
20nM
50nM
100nM
200nM
500nM
1000nM
