# Supplementary material for: Directed evolution of a stem-helix–targeting antibody enables MERS-CoV cross-neutralization through enhanced binding affinity
Source: PLoS Pathog. 2026 Aug 7;22(8):e1014393. doi: 10.1371/journal.ppat.1014393 (PMC13480645; doi:10.1371/journal.ppat.1014393)

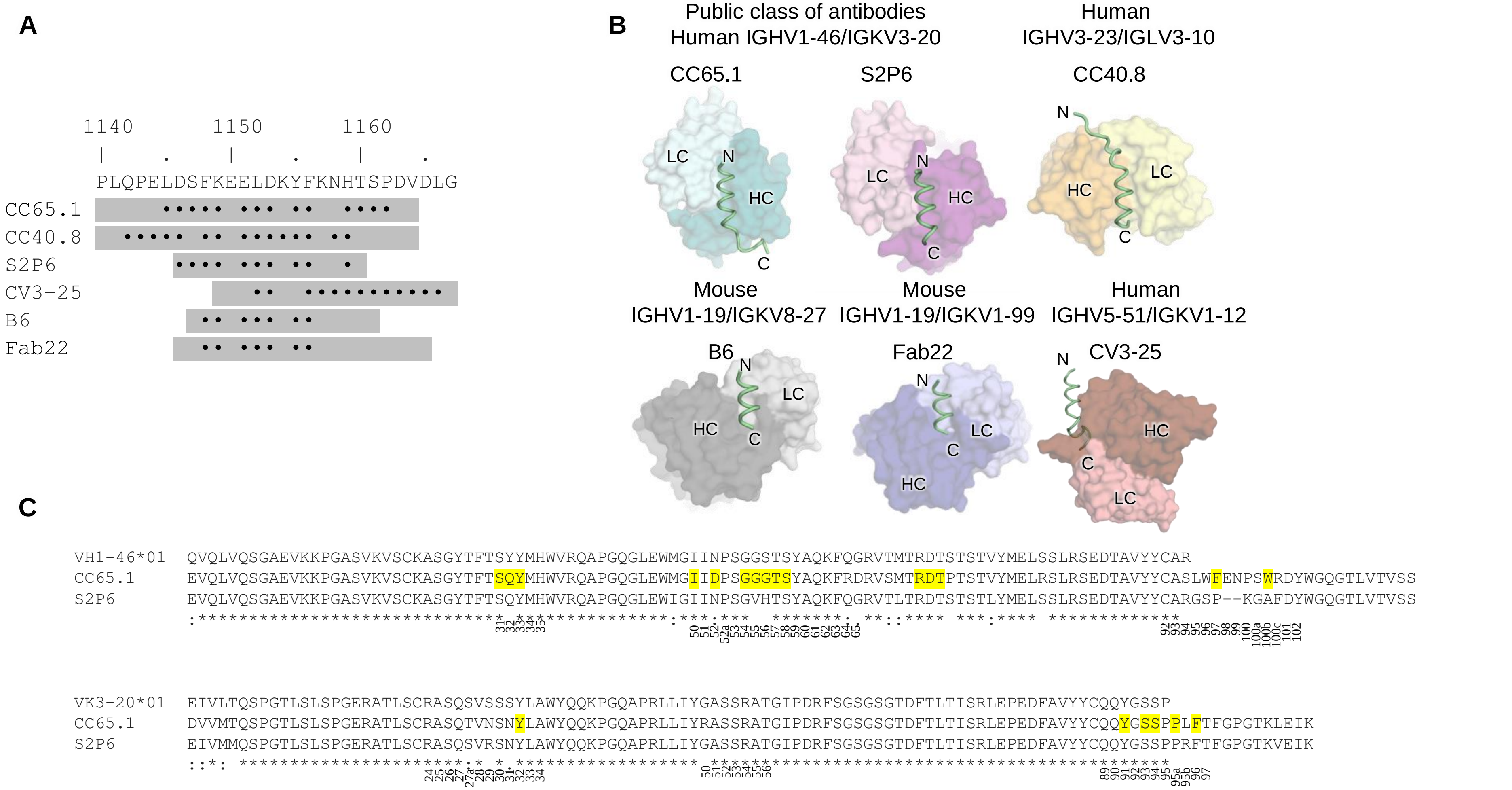

D

%change with WT<70%
IC_{50} fold change>5

SARS-CoV-2	BLI response	%change with WT	IC_{50}(μg/mL)	IC_{50} fold change
WT	3.21	100.0%	6.47	1.0
P1140A	3.21	99.8%	1.55	0.2
L1141A	3.11	96.9%	0.42	0.1
Q1142A	3.12	97.1%	4.26	0.7
P1143A	3.13	97.6%	12.18	1.9
E1144A	3.13	97.5%	13.28	2.1
L1145A	3.08	95.7%	N.A.	N.A.
D1146A	3.04	94.5%	78.37	12.1
S1147A	2.71	84.3%	17.96	2.8
F1148A	1.82	56.5%	>300	>40
K1149A	2.87	89.5%	0.48	0.1
E1150A	3.06	95.2%	5.28	0.8
E1151A	2.36	73.4%	11.99	1.9
L1152A	2.50	77.8%	39.56	6.1
D1153A	2.82	87.7%	25.52	3.9
K1154A	2.87	89.3%	0.35	0.1
Y1155A	2.56	79.7%	>300	>40
F1156A	1.09	34.0%	>300	>40
K1157A	2.92	90.8%	0.76	0.1
N1158A	2.68	83.5%	0.77	0.1
H1159A	2.72	84.7%	1.75	0.3
T1160A	2.94	91.6%	0.53	0.1
S1161A	3.03	94.3%	1.40	0.2
P1162A	2.80	87.3%	5.05	0.8
D1163A	2.69	83.8%	15.53	2.4
V1164A	2.51	78.1%	4.74	0.7

IC_{50} (μg/mL)	<1	1-10	10-300	>300

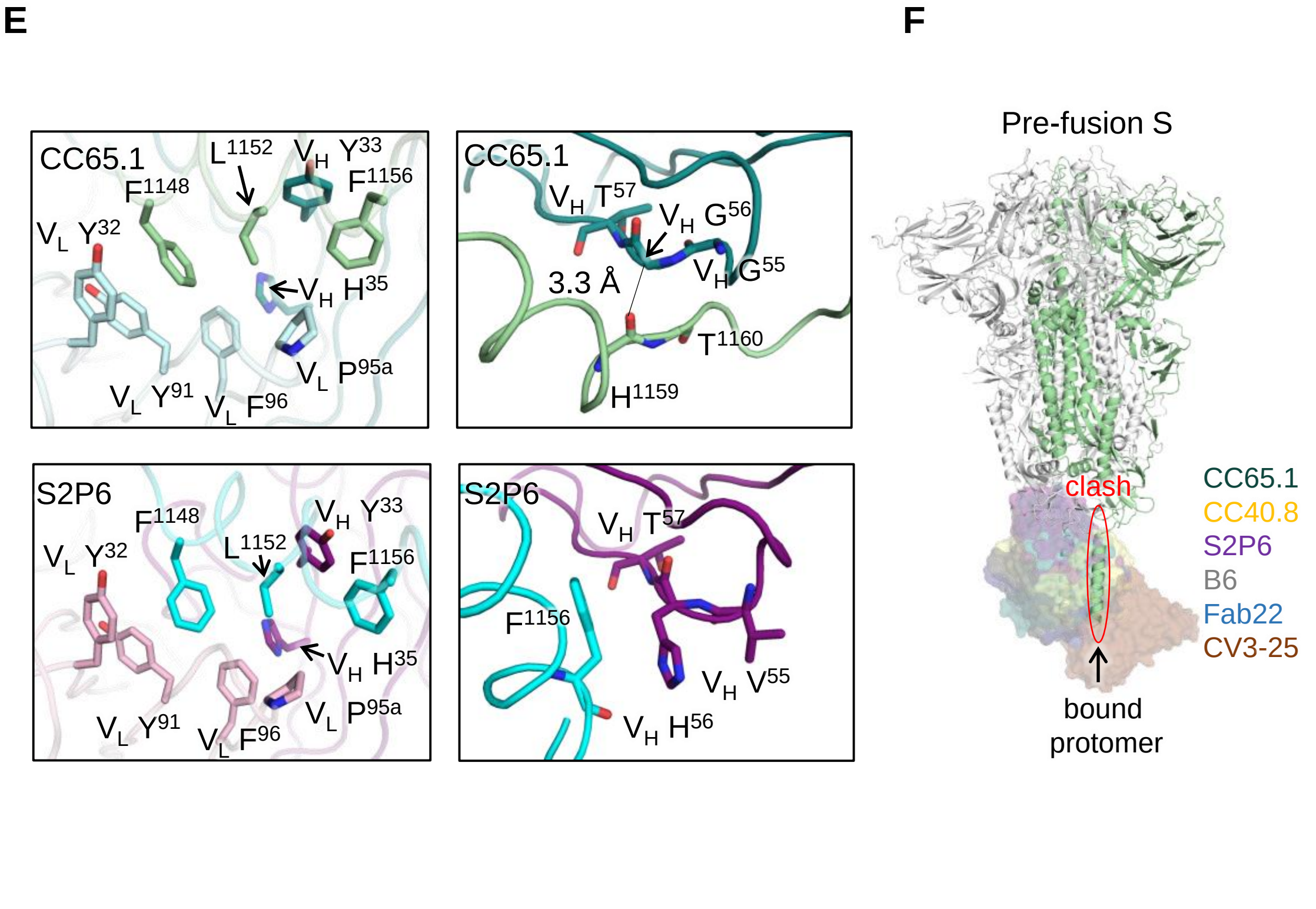

Supplement: Supplementary file 2 [file ppat.1014393.s002.pdf]
