# Supplementary material for: Directed evolution of a stem-helix–targeting antibody enables MERS-CoV cross-neutralization through enhanced binding affinity
Source: PLoS Pathog. 2026 Aug 7;22(8):e1014393. doi: 10.1371/journal.ppat.1014393 (PMC13480645; doi:10.1371/journal.ppat.1014393)
Supplement: Supplementary file 3 [file ppat.1014393.s003.pdf]

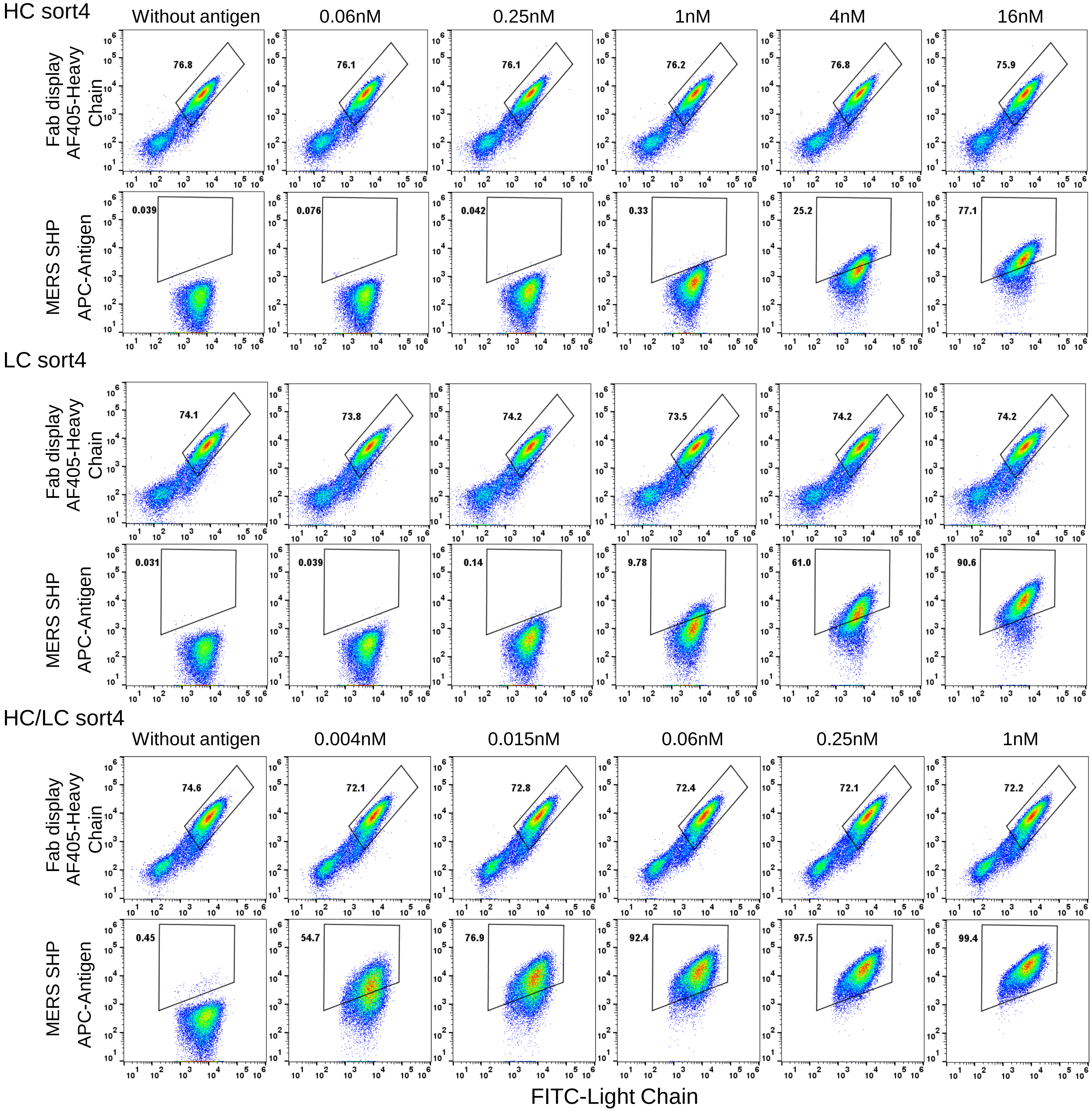

HC sort4
Without antigen
0.06nM
0.25nM
1nM
4nM
16nM
Fab display AF405-Heavy Chain
76.8
76.1
76.1
76.2
76.8
75.9
MERS SHP APC-Antigen
0.039
0.076
0.042
0.33
25.2
77.1
LC sort4
Fab display AF405-Heavy Chain
74.1
73.8
74.2
73.5
74.2
74.2
MERS SHP APC-Antigen
0.031
0.039
0.14
9.78
61.0
90.6
HC/LC sort4
Without antigen
0.004nM
0.015nM
0.06nM
0.25nM
1nM
Fab display AF405-Heavy Chain
74.6
72.1
72.8
72.4
72.1
72.2
MERS SHP APC-Antigen
0.45
54.7
76.9
92.4
97.5
99.4
FITC-Light Chain
