# Supplementary material for: Directed evolution of a stem-helix–targeting antibody enables MERS-CoV cross-neutralization through enhanced binding affinity
Source: PLoS Pathog. 2026 Aug 7;22(8):e1014393. doi: 10.1371/journal.ppat.1014393 (PMC13480645; doi:10.1371/journal.ppat.1014393)
Supplement: Supplementary file 4 [file ppat.1014393.s004.pdf]

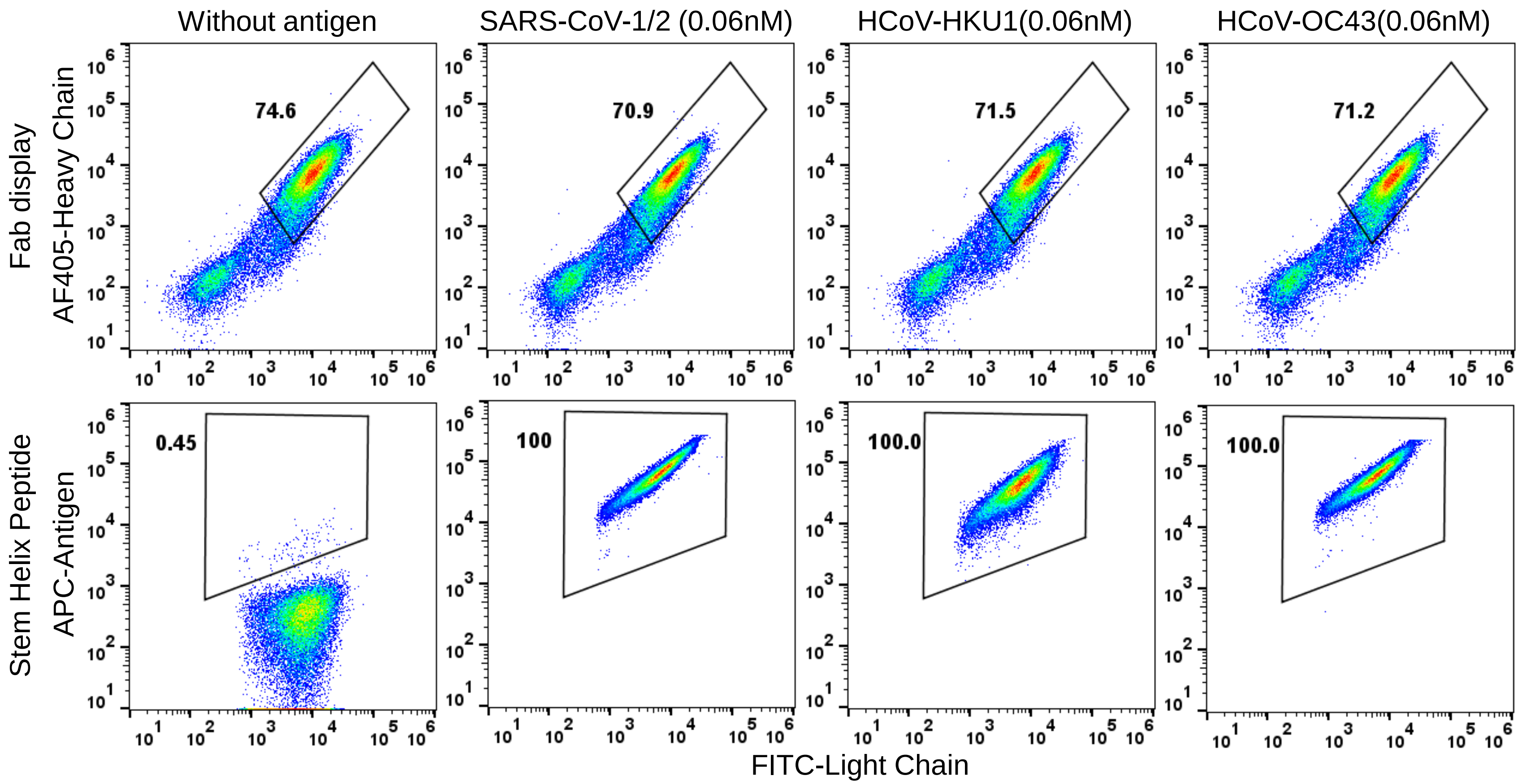

Without antigen
SARS-CoV-1/2 (0.06nM)
HCoV-HKU1(0.06nM)
HCoV-OC43(0.06nM)
Fab display
AF405-Heavy Chain
74.6
70.9
71.5
71.2
Stem Helix Peptide
APC-Antigen
0.45
100
100.0
100.0
FITC-Light Chain
