# Supplementary material for: Directed evolution of a stem-helix–targeting antibody enables MERS-CoV cross-neutralization through enhanced binding affinity
Source: PLoS Pathog. 2026 Aug 7;22(8):e1014393. doi: 10.1371/journal.ppat.1014393 (PMC13480645; doi:10.1371/journal.ppat.1014393)
Supplement: Supplementary file 5 [file ppat.1014393.s005.pdf]

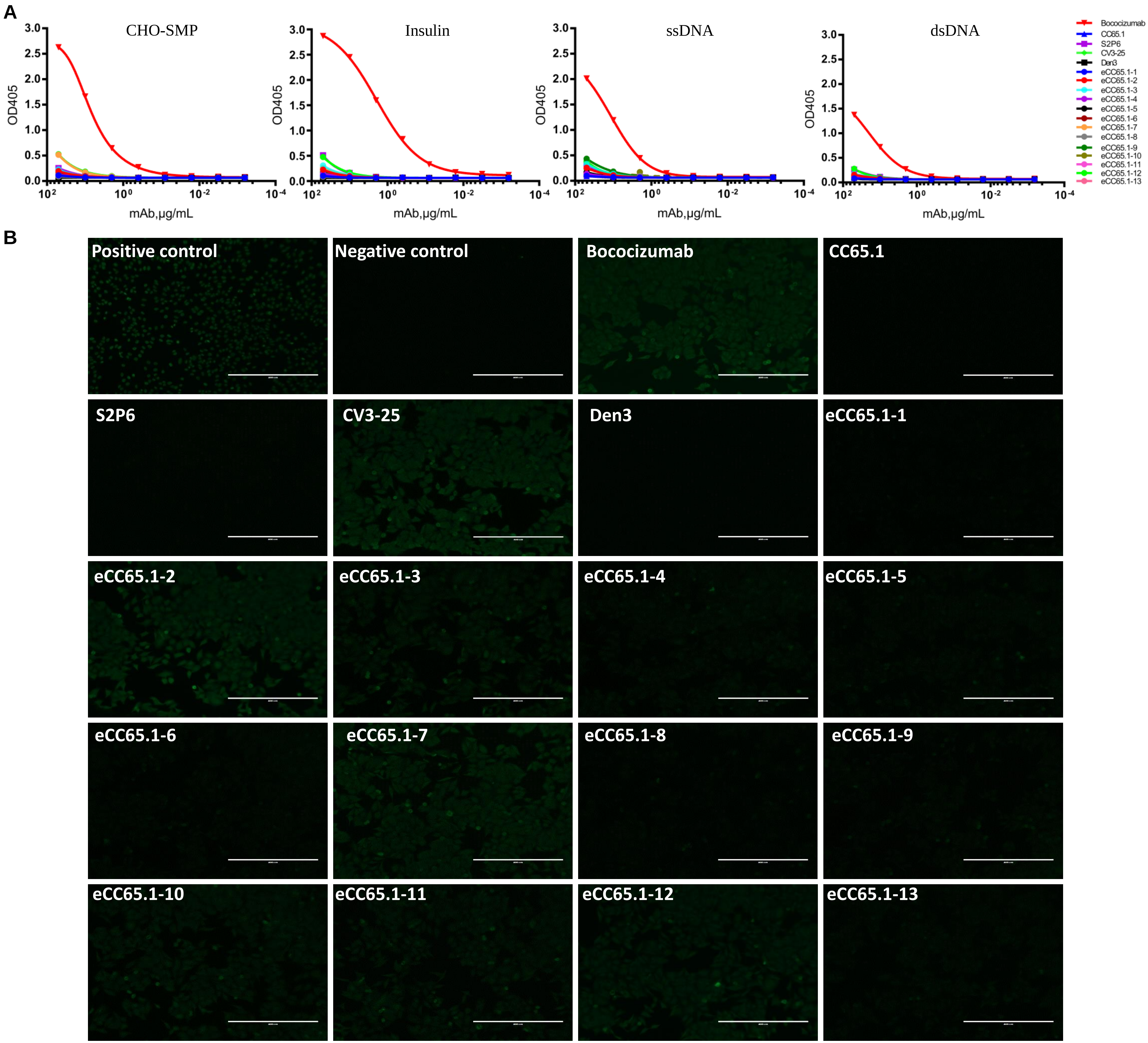
A
CHO-SMP
Insulin
ssDNA
dsDNA
OD405
mAb,μg/mL
Bococizumab
CC65.1
S2P6
CV3-25
Den3
eCC65.1-1
eCC65.1-2
eCC65.1-3
eCC65.1-4
eCC65.1-5
eCC65.1-6
eCC65.1-7
eCC65.1-8
eCC65.1-9
eCC65.1-10
eCC65.1-11
eCC65.1-12
eCC65.1-13
B
Positive control
Negative control
Bococizumab
CC65.1
S2P6
CV3-25
Den3
eCC65.1-1
eCC65.1-2
eCC65.1-3
eCC65.1-4
eCC65.1-5
eCC65.1-6
eCC65.1-7
eCC65.1-8
eCC65.1-9
eCC65.1-10
eCC65.1-11
eCC65.1-12
eCC65.1-13
