# Supplementary material for: Directed evolution of a stem-helix–targeting antibody enables MERS-CoV cross-neutralization through enhanced binding affinity
Source: PLoS Pathog. 2026 Aug 7;22(8):e1014393. doi: 10.1371/journal.ppat.1014393 (PMC13480645; doi:10.1371/journal.ppat.1014393)

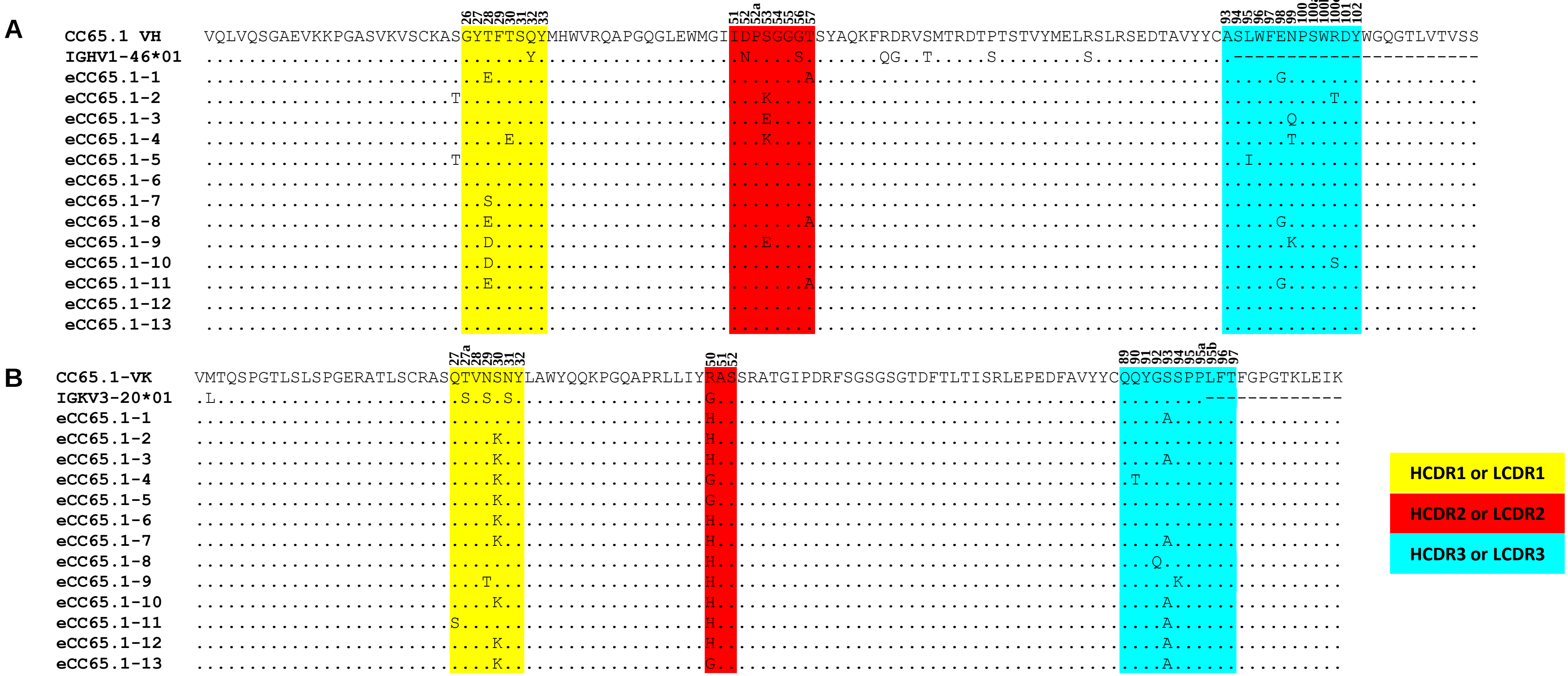

C

		S2P6	CC65.1	eCC65.1-1	eCC65.1-2	eCC65.1-3	eCC65.1-4	eCC65.1-5	eCC65.1-6	eCC65.1-7	eCC65.1-8	eCC65.1-9	eCC65.1-10	eCC65.1-11	eCC65.1-12	eCC65.1-13
IC_{50} (μg/mL)	SARS-CoV-2	6.16	6.47	5.79	2.05	4.67	1.84	1.63	2.98	3.01	3.95	2.60	1.86	5.26	5.77	2.41
	SARS-CoV-1	2.98	4.05	6.83	3.76	5.00	2.02	1.47	5.02	4.17	6.28	2.28	1.49	4.32	1.49	0.67
	MERS-CoV	8.37	>300	32.61	2.50	2.59	14.28	9.88	6.46	3.60	152.70	10.16	5.45	25.62	4.18	23.17
SARS-CoV-2 Spike	Response	0.98	0.78	0.74	0.83	0.83	0.86	0.84	0.81	0.81	0.75	0.85	0.80	0.73	0.80	0.78
	KD^{APP} (M)	1.30E-09	2.72E-10	8.43E-10	<1.0E-12	<1.0E-12	1.36E-11	<1.0E-12	4.72E-11	1.57E-10	9.45E-10	1.02E-11	<1.0E-12	8.01E-10	<1.0E-12	8.19E-11
	kon(1/Ms)	5.59E+05	2.81E+05	2.36E+05	2.83E+05	2.89E+05	2.87E+05	2.86E+05	2.61E+05	2.70E+05	2.58E+05	3.06E+05	2.78E+05	2.49E+05	2.73E+05	2.56E+05
	koff(1/Ms)	7.26E-04	7.64E-05	1.99E-04	<1.00E-7	<1.00E-7	3.91E-06	<1.00E-7	1.23E-05	4.24E-05	2.44E-04	3.12E-06	<1.00E-7	1.99E-04	<1.00E-7	2.09E-05
SARS-CoV-2 SHP	Response	4.54	3.21	3.16	3.39	3.29	3.74	3.45	3.37	3.35	3.19	3.50	3.07	3.15	3.27	3.33
	KD^{APP} (M)	4.84E-10	1.73E-10	6.37E-11	1.11E-10	9.76E-11	5.10E-11	1.16E-10	7.38E-11	1.18E-10	1.52E-10	1.98E-10	1.06E-10	8.76E-11	6.13E-11	1.01E-10
	kon(1/Ms)	2.15E+05	2.93E+05	3.08E+05	2.99E+05	3.06E+05	3.97E+05	3.16E+05	3.23E+05	2.94E+05	2.88E+05	2.97E+05	2.97E+05	3.16E+05	3.44E+05	3.10E+05
	koff(1/Ms)	1.04E-04	5.07E-05	1.96E-05	3.33E-05	2.98E-05	2.02E-05	3.66E-05	2.38E-05	3.47E-05	4.38E-05	5.87E-05	3.13E-05	2.77E-05	2.11E-05	3.13E-05
MERS-CoV Spike	Response	0.79	0.21	0.20	0.38	0.30	0.33	0.33	0.28	0.32	0.26	0.40	0.28	0.21	0.33	0.26
	KD^{APP} (M)	6.26E-09	2.76E-08	1.93E-08	1.59E-08	1.72E-08	1.17E-08	1.52E-08	1.52E-08	1.80E-08	1.26E-08	1.37E-08	1.87E-08	1.84E-08	1.01E-08	1.42E-08
	kon(1/Ms)	2.26E+05	1.33E+05	1.02E+05	1.11E+05	8.70E+04	1.47E+05	1.17E+05	1.24E+05	9.98E+04	1.38E+05	1.14E+05	9.78E+04	1.11E+05	1.24E+05	1.38E+05
	koff(1/Ms)	1.41E-03	3.66E-03	1.96E-03	1.76E-03	1.49E-03	1.72E-03	1.79E-03	1.89E-03	1.80E-03	1.75E-03	1.56E-03	1.83E-03	2.04E-03	1.25E-03	1.95E-03
MERS-CoV SHP	Response	4.81	3.16	3.44	3.71	3.41	4.00	3.68	3.51	3.78	3.49	3.86	3.27	3.27	3.57	3.46
	KD^{APP} (M)	7.23E-10	4.82E-09	5.89E-10	4.01E-10	2.62E-10	9.20E-10	9.01E-10	7.10E-10	4.22E-10	1.24E-09	8.21E-10	3.62E-10	5.57E-10	3.71E-10	7.22E-10
	kon(1/Ms)	3.15E+05	4.04E+05	3.42E+05	3.12E+05	3.03E+05	4.35E+05	3.15E+05	3.38E+05	3.32E+05	3.07E+05	3.32E+05	2.93E+05	3.15E+05	3.49E+05	3.09E+05
	koff(1/Ms)	2.27E-04	1.95E-03	2.01E-04	1.25E-04	7.94E-05	4.00E-04	2.83E-04	2.40E-04	1.40E-04	3.80E-04	2.73E-04	1.06E-04	1.75E-04	1.29E-04	2.23E-04

IC_{50} (μg/mL)
<1
1-10
10-300
>300

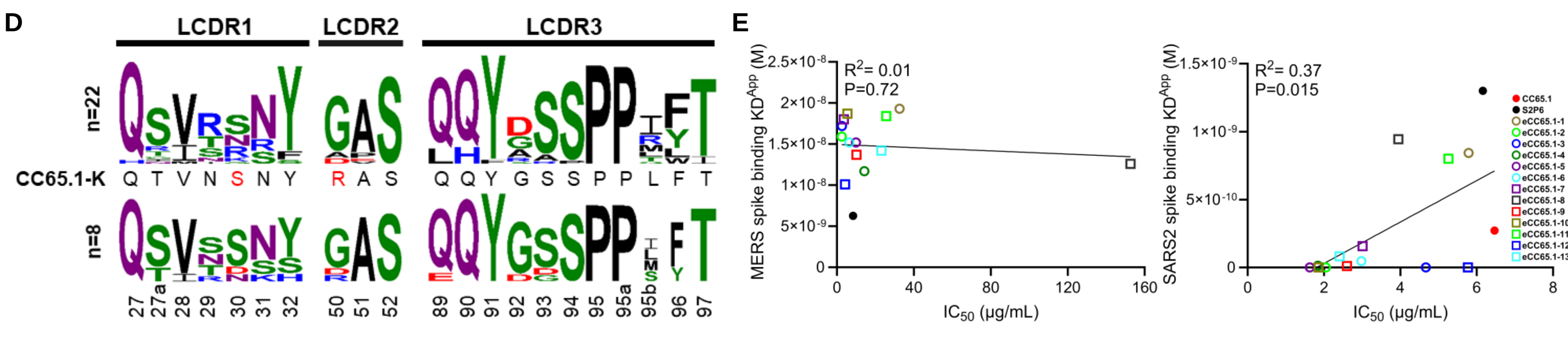

Supplement: Supplementary file 6 [file ppat.1014393.s006.pdf]
