# Supplementary material for: Directed evolution of a stem-helix–targeting antibody enables MERS-CoV cross-neutralization through enhanced binding affinity
Source: PLoS Pathog. 2026 Aug 7;22(8):e1014393. doi: 10.1371/journal.ppat.1014393 (PMC13480645; doi:10.1371/journal.ppat.1014393)
Supplement: Supplementary file 7 [file ppat.1014393.s007.pdf]

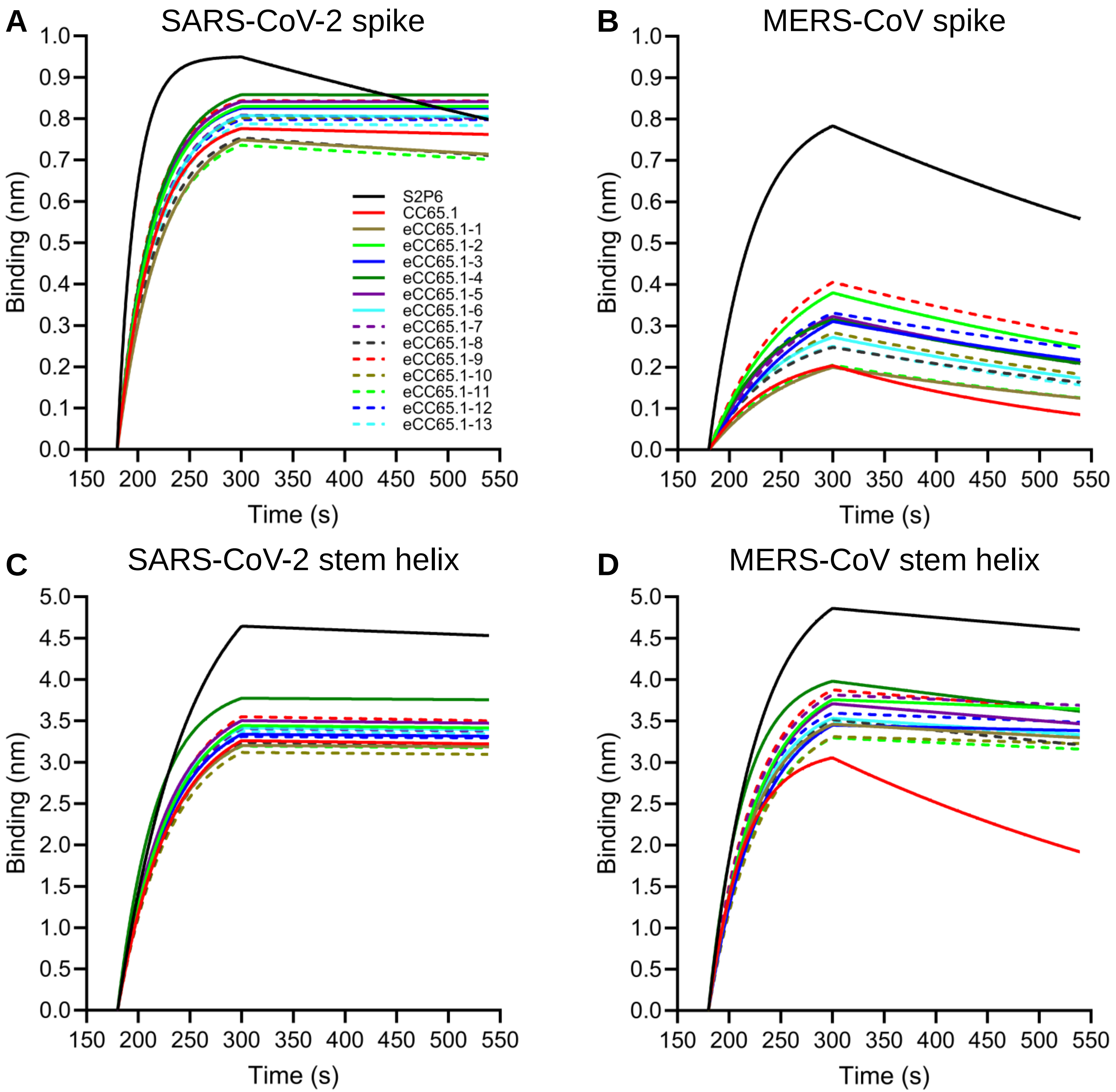

A
SARS-CoV-2 spike
B
MERS-CoV spike
C
SARS-CoV-2 stem helix
D
MERS-CoV stem helix
Binding (nm)
Time (s)
S2P6
CC65.1
eCC65.1-1
eCC65.1-2
eCC65.1-3
eCC65.1-4
eCC65.1-5
eCC65.1-6
eCC65.1-7
eCC65.1-8
eCC65.1-9
eCC65.1-10
eCC65.1-11
eCC65.1-12
eCC65.1-13
