# Supplementary material for: Directed evolution of a stem-helix–targeting antibody enables MERS-CoV cross-neutralization through enhanced binding affinity
Source: PLoS Pathog. 2026 Aug 7;22(8):e1014393. doi: 10.1371/journal.ppat.1014393 (PMC13480645; doi:10.1371/journal.ppat.1014393)
Supplement: Supplementary file 8 [file ppat.1014393.s008.pdf]

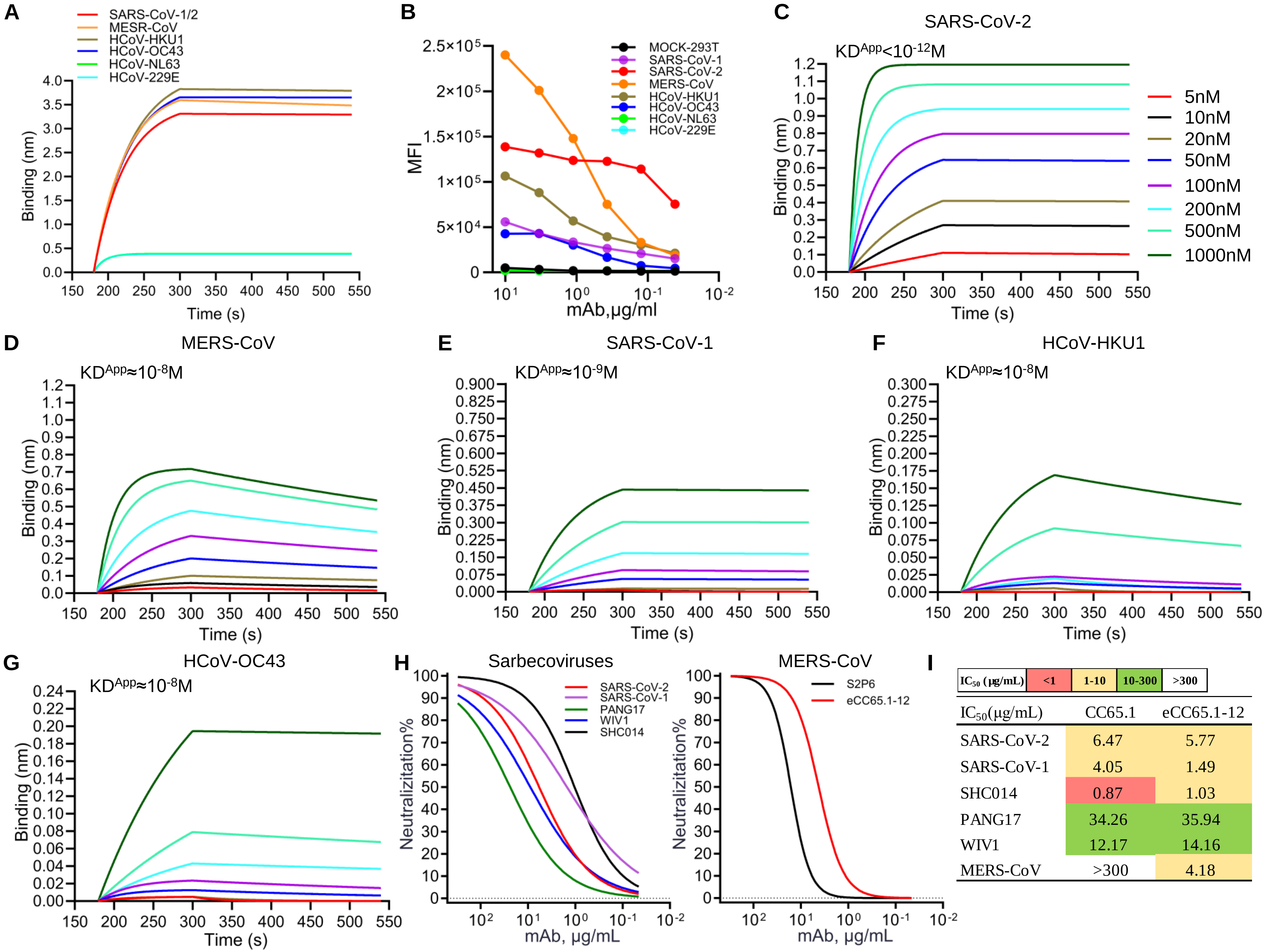


| IC50(μg/mL) | CC65.1 | eCC65.1-12 |
|---|---|---|
| SARS-CoV-2 | 6.47 | 5.77 |
| SARS-CoV-1 | 4.05 | 1.49 |
| SHC014 | 0.87 | 1.03 |
| PANG17 | 34.26 | 35.94 |
| WIV1 | 12.17 | 14.16 |
| MERS-CoV | >300 | 4.18 |
