# Supplementary material for: Directed evolution of a stem-helix–targeting antibody enables MERS-CoV cross-neutralization through enhanced binding affinity
Source: PLoS Pathog. 2026 Aug 7;22(8):e1014393. doi: 10.1371/journal.ppat.1014393 (PMC13480645; doi:10.1371/journal.ppat.1014393)
Supplement: Supplementary file 9 [file ppat.1014393.s009.pdf]

		MFI						Spike			Stem helix peptides		
	**mAb(μg/ml)**	**10.00**	**3.33**	**1.11**	**0.37**	**0.12**	**0.04**	**KD^{App} (M)**	**kon (1/Ms)**	**koff (1/Ms)**	**KD^{App} (M)**	**kon (1/Ms)**	**koff (1/Ms)**
**CC65.1**	**MOCK-293T**	1474	1579	1430	1534	1493	1376		N.D.			N.D.	
	**SARS-CoV-1**	42857	34906	26742	22798	18074	11906	8.20E-10	4.32E+05	3.54E-04	1.73E-10	2.93E+05	5.07E-05
	**SARS-CoV-2**	144905	127685	124789	119288	102680	63400	2.72E-10	2.81E+05	7.64E-05			
	**MERS-CoV**	20548	11837	6773	3893	2549	2003	2.76E-08	1.33E+05	3.66E-03	4.82E-09	4.04E+05	1.95E-03
	**HCoV-HKU1**	10345	8964	7060	5233	3839	2979	2.12E-08	1.16E+05	2.46E-03	8.67E-10	2.45E+05	2.13E-04
	**HCoV-OC43**	31898	35505	19859	8288	4749	3280	3.08E-08	5.77E+04	1.77E-03	1.24E-10	2.93E+05	3.62E-05
	**HCoV-NL63**	1640	1623	1623	1651	1621	1613		N.D.			N.D.	
	**HCoV-229E**	1642	1620	1603	1634	1624	1621		N.D.			N.D.	
**CC65.1-12**	**MOCK-293T**	4793	3251	1759	1533	1430	1407		N.D.			N.D.	
	**SARS-CoV-1**	55745	42960	33492	26370	20731	15207	2.03E-09	5.26E+04	1.07E-04	6.13E-11	3.44E+05	2.11E-05
	**SARS-CoV-2**	138682	131730	123676	122520	114021	75275	<1.00E-12	2.73E+05	<1.00E-7			
	**MERS-CoV**	240163	200902	147890	75072	32996	18774	1.01E-08	1.24E+05	1.25E-03	3.71E-10	3.49E+05	1.29E-04
	**HCoV-HKU1**	106435	87983	56750	39216	30306	21206	5.39E-08	7.34E+04	3.95E-03	1.29E-10	3.01E+05	3.90E-05
	**HCoV-OC43**	42606	42796	30132	16550	7461	4519	2.92E-08	6.94E+04	2.03E-03	1.63E-11	3.26E+05	5.31E-06
	**HCoV-NL63**	2009	1877	1715	1681	1623	1613		N.D.			N.D.	
	**HCoV-229E**	2096	1834	1732	1680	1615	1627		N.D.			N.D.	
