# Supplementary material for: Directed evolution of a stem-helix–targeting antibody enables MERS-CoV cross-neutralization through enhanced binding affinity
Source: PLoS Pathog. 2026 Aug 7;22(8):e1014393. doi: 10.1371/journal.ppat.1014393 (PMC13480645; doi:10.1371/journal.ppat.1014393)
Supplement: Supplementary file 10 [file ppat.1014393.s010.pdf]

Data collection			
	CC65.1 scFv + SARS-CoV-2 S2 stem peptide	CC65.1 Fab + MERS-CoV S2 stem peptide	eCC65.1-12 Fab + MERS-CoV S2 stem peptide
Beamline	SSRL 12-1	ALS 5.0.1	APS23-ID-B
Wavelength (Å)	0.97946	0.97741	0.97930
Space group	$C\ 2\ 2\ 2_1$	C 2	C 2
Unit cell parameters			
a, b, c (Å)	83.5, 98.5, 144.0	95.4, 58.6, 96.7	91.0, 60.0, 81.8
α, β, γ (°)	90, 90, 90	90, 93.5, 90	90, 98.0, 90
Resolution (Å) [a]	50.0–2.05 (2.09–2.05)	50.0–2.70 (2.83–2.70)	50.0–2.51 (2.55–2.51)
Unique reflections [a]	34,344 (2,176)	14,853 (1,482)	13,990 (913)
Redundancy [a]	7.0 (2.8)	6.5 (6.7)	3.9 (3.4)
Completeness (%) [a]	91.4 (55.5)	100 (100)	99.5 (96.3)
$<I/\sigma_I>$ [a]	21.0 (4.4)	5.0 (1.4)	8.7 (1.1)
R_{sym}[b] (%) [a]	13.4 (24.8)	29.3 (>100)	14.2 (>100)
R_{pim}[b] (%) [a]	5.1 (16.0)	18.8 (78.7)	8.0 (59.3)
$CC_{1/2}$[c] (%) [a]	98.4 (92.6)	97.5 (63.6)	97.5 (50.0)
**Refinement statistics**			
Resolution (Å)	40.1–2.05	48.3–2.70	44.0–2.51
Reflections (work)	34,341	14,833	13,988
Reflections (test)	2,177	1,479	913
R_{cryst}[d] / R_{free}[e] (%)	19.2/22.9	25.0/30.6	21.8/26.4
No. of atoms	4,375	3,562	3,600
Antibodies	3,562	3,314	3,353
Peptides	322	141	113
Other (SO4)	0	20	25
Solvent	491	87	109
Average *B*-value (Å²)	26	29	43
Antibodies	24	29	42
Peptide	38	38	59
Other (SO4)	N/A	36	73
Solvent	31	26	37
Wilson *B*-value (Å²)	21	33	43
**RMSD from ideal geometry**			
Bond length (Å)	0.002	0.003	0.02
Bond angle (°)	0.55	0.71	0.58
**Ramachandran statistics**[f]			
Favored	97.5	93.7	96.0
Outliers	0	0	0
**PDB code**	9YJI	9YJJ	9YJK

[a] Numbers in parentheses refer to the highest resolution shell.

[b] $R_{sym} = \Sigma_{hkl} \Sigma_i | I_{hkl,i} - <I_{hkl}> | / \Sigma_{hkl} \Sigma_i I_{hkl,i}$ and $R_{pim} = \Sigma_{hkl} (1/(n-1))^{1/2} \Sigma_i | I_{hkl,i} - <I_{hkl}> | / \Sigma_{hkl} \Sigma_i I_{hkl,i}$, where $I_{hkl,i}$ is the scaled intensity of the ith measurement of reflection h, k, l, $<I_{hkl}>$ is the average intensity for that reflection, and *n* is the redundancy.

[c] $CC_{1/2}$ = Pearson correlation coefficient between two random half datasets.

[d] $R_{cryst} = \Sigma_{hkl} | F_o - F_c | / \Sigma_{hkl} | F_o |$ x 100, where F_o and F_c are the observed and calculated structure factors, respectively.

[e] R_{free} was calculated as for R_{cryst}, but on a test set comprising 5% of the data excluded from refinement.

[f] From MolProbity [52].
