# Supplementary material for: Directed evolution of a stem-helix–targeting antibody enables MERS-CoV cross-neutralization through enhanced binding affinity
Source: PLoS Pathog. 2026 Aug 7;22(8):e1014393. doi: 10.1371/journal.ppat.1014393 (PMC13480645; doi:10.1371/journal.ppat.1014393)
Supplement: Supplementary file 11 [file ppat.1014393.s012.docx]

**Responses to the Reviewers’ comments**

PPATHOGENS-D-25-02460

**Directed evolution of a stem-helix–targeting antibody enables MERS-CoV cross-neutralization through enhanced binding affinity**

**Reviewer #1:** The authors describe the isolation of CC65.1, a stem-helix targeting antibody, and its subsequent affinity maturation to generate variants (e.g., eCC65.1) capable of neutralizing MERS-CoV. The study is well-conceived, and the use of directed evolution to broaden the neutralizing breadth of antibodies targeting cryptic epitopes is of high interest to the field. The manuscript is well-written, and the structural analyses provided are robust. However, a few technical details regarding the spike constructs and mechanism of action need clarification to ensure a comprehensive understanding of the discovery.

**Response:** We thank the reviewer for the positive and thoughtful assessment of our study.

Part II – Major Issues: Key Experiments Required for Acceptance

Please use this section to detail the key new experiments or modifications of existing experiments that should be absolutely required to validate study conclusions.

Generally, there should be no more than 3 such required experiments or major modifications for a "Major Revision" recommendation. If more than 3 experiments are necessary to validate the study conclusions, then you are encouraged to recommend "Reject".

**Reviewer #1:** 1. Mechanism of Neutralization: The manuscript lacks a discussion on how affinity maturation enables neutralization of MERS-CoV when the parent antibody binds but does not neutralize. The current explanation treats affinity as a standalone metric. The authors should explicitly connect their hypothesis on accessibility (Lines 258-260) to the neutralization data. Does the high affinity of eCC65.1 allow it to "catch" the transiently exposed stem helix on MERS-CoV more effectively, thereby increasing occupancy and blocking the hairpin formation in the fusion machinery? This complexity of epitope accessibility is further highlighted by the SARS-CoV-1 data. Lines 121-122 claim "stronger binding" for SARS-CoV-1 in "both formats," yet CELISA data (Fig S1B) shows significantly weaker binding for SARS-CoV-1 compared to SARS-CoV-2, despite identical epitope sequences. Discussing this "occupancy model" is necessary to resolve the apparent paradox of why the parent antibody binds but fails to neutralize.

**Response:** We thank the reviewer for this insightful comment. We agree that binding affinity alone does not fully explain the acquisition of MERS-CoV neutralization and have revised the manuscript to incorporate the potential for an accessibility-dependent “occupancy model” being operational here. Structural analysis indicates that the S2 stem helix epitope is partially occluded in the prefusion spike trimer (Fig S2F) and becomes transiently exposed during the fusion process, particularly in prehairpin intermediates. Recent in situ cryo-ET studies of SARS-CoV-2 (https://doi.org/10.1126/science.adn5658; https://doi.org/10.1038/s41467-025-60406-z) have demonstrated that stem helix antibodies neutralize by engaging these transiently exposed intermediates and blocking HR2-mediated back-zippering, thereby arresting spike refolding and membrane fusion.

In this context, neutralization depends on the ability of a stem helix antibody to achieve sufficient epitope occupancy during these short-lived exposure windows to some extent. The parental CC65.1 antibody binds to the MERS-CoV stem helix but with substantially reduced binding affinity and a faster dissociation rate (koff) (Fig S6C and S7D), which limits its ability to effectively capture and retain the transiently exposed epitope to block formation of the postfusion hairpin, thereby resulting in a lack of MERS-CoV neutralization. In contrast, in vitro affinity maturation of CC65.1 (eCC65.1 variants) significantly reduces the dissociation rate and enhances binding affinity (Fig S6C and S7D), potentially enabling more efficient engagement and sustained occupancy of the transiently exposed stem helix. This “occupancy model” is further supported by the observation that neutralization potency is more likely to have a positive correlation with binding affinity to stem helix peptide, especially for MERS-CoV (Fig 3F and S6E).

Notably, SARS-CoV-1 and SARS-CoV-2 share an identical stem helix sequence, suggesting that intrinsic epitope binding affinity and capture efficiency of both CC65.1 and eCC65.1 variants to transiently exposed stem helix are likely similar between these two viruses. Despite this, measurable differences in binding to SARS-CoV-1 versus SARS-CoV-2 spikes are observed across both cell surface-expressed spikes and S-2P proteins (Fig 1F, S1B-C and S8B, C, E), with weaker binding to SARS-CoV-1. However, neutralization potency of these antibodies to SARS-CoV-1 and SARS-CoV-2 remains comparable (Fig S6C), indicating that intrinsic epitope affinity, rather than bulk spike binding, governs neutralization when the accessibility of stem helix is limiting. This also highlights the potential differences between the S-2P or cell surface-expressed spike proteins versus the spike proteins expressed on the surface of infectious virions.

Together, these findings support a model in which affinity maturation enhances neutralization by increasing binding affinity, and which improves the probability and duration of antibody engagement with a transiently accessible epitope, thereby enabling effective inhibition of the fusion machinery.

As per the suggestion of the reviewer, we added/updated some descriptions and comments in the “Introduction Lines 82-86”, “Result Lines 253-256” and “Discussion Lines 349-354, 356-360” section, respectively.

**“Introduction” section**

Lines 82-86: “Recent in situ cryo-ET studies of SARS-CoV-2 show that stem-helix epitopes are only partially exposed on the prefusion spike, and that the bnAbs targeting this site likely neutralize by engaging the transiently exposed stem-helix during viral entry and blocking HR2-mediated back-zippering, thereby arresting spike refolding and membrane fusion.”

**“Result” section**

Lines 253-256: “All of the affinity-matured antibodies bind the MERS-CoV stem helix peptide with higher affinity than the parental CC65.1 antibody, showing an average 9.1-fold increase in KD^APP^ values (range: 3.9-18.4-fold) (Fig 3C, S6C and S7D). This affinity improvement is primarily driven by a reduced dissociation rate (koff) (Fig S6C and S7D).”

**“Discussion” section**

Lines 349-354: “Structural and biochemical analyses reveal that the lack of MERS-CoV neutralization is attributable to reduced binding affinity and fast dissociation rate (koff) of CC65.1 to MERS-CoV stem helix, which may be driven in part by sequence divergence at the N-terminal end of the S2 stem helix, thereby limiting its ability to effectively capture and retain the transiently exposed MERS-CoV stem helix to block formation of the postfusion hairpin and membrane fusion.”

Lines 356-360: “The engineered variants, for example eCC65.1-12, acquire substantially enhanced binding affinity and reduced dissociation rate to MERS-CoV stem helix peptide, which may enable more efficient engagement and sustained occupancy of the transiently exposed MERS-CoV epitope, resulting in acquiring MERS-CoV-neutralizing activity while retaining broad reactivity to other betacoronaviruses.”

2. Clarification of Spike Constructs: The text fails to explicitly state whether WT, S-2P, or HexaPro constructs were used for the specific spike proteins (SARS-CoV-1/2 vs. MERS-CoV) in the binding assays (SPR/BLI). Please provide these details in the Methods section.

**Response:** Apologies for this oversight in the initial submission. All of the spike proteins used for BLI, ELISA and B cell sorting are S-2P constructs. As per the reviewer’s suggestion, we added the S-2P construct details under the “Expression and purification of betacoronavirus recombinant soluble spike protein and antibody” section of “Materials and methods”, and replaced the description, such as “spike” and “recombinant soluble spike” by “S-2P” at the appropriate positions in the manuscript.

Lines 484-491: “To generate stable trimeric spike proteins, double proline (2P) substitutions were introduced into the S2 subunit at the following positions: K968/V969 in SARS-CoV-1, K986/V987 in SARS-CoV-2, V1060/L1061 in MERS-CoV, A1071/L1072 in HCoV-HKU1, and A1078/L1079 in HCoV-OC43. In addition, the furin cleavage sites of spikes (SARS-CoV-1: 664–667; SARS-CoV-2: 682–685; MERS-CoV: 748–751; HCoV-HKU1: 756–760 and HCoV-OC43: 762–766) were replaced with a “GSAS” linker. The trimerization domain of T4 fibritin was appended to the C-terminus, followed by an HRV-3C protease cleavage site, a 6×His tag, and an AviTag, each separated by a GS linker, to facilitate purification and biotinylation.”

Lines 112-113, 118, 126, 140, and so on (31 in total): Changed to “S-2P”.

3. Terminology: The authors frequently use the term "rational engineering." However, the approach utilized is directed evolution, not rational design. In a typical rational design approach (e.g., structure-guided), one would target the direct interface residues to improve affinity. In contrast, the key impact here appears to come from paratope stabilization between CDRL1 and L2, potentially reducing the entropic cost of capturing a transiently breathing epitope. Notably, the interacting residues are effectively the same (with the exception of the S93A mutation), while the significant improvements arise from distal stabilization (e.g., the Lys-His interaction stabilizing the Tyr rotamer). I recommend reframing the text to highlight that directed evolution was capable of identifying this sophisticated, non-intuitive structural solution, emphasizing the power of the method used.

**Response:** We appreciate the reviewer’s thoughtful and constructive comment. We agree that the terms “rational antibody engineering” and “rational engineering” were not appropriately used in our manuscript. Our approach is based more on a directed evolution strategy (SAMPLER) rather than classical structure-guided rational design. But as highlighted in the “Author Summary” (Lines 59-61), our primary goal was to emphasize the role of “in vitro affinity maturation” in expanding antibody neutralization breadth. To better reflect this conceptual focus, we have replaced “rational antibody engineering” and “rational engineering” by “in vitro affinity maturation” in the manuscript (Lines 44, 95, 372 and 383, 4 in total).

Part III – Minor Issues: Editorial and Data Presentation Modifications

Please use this section for editorial suggestions as well as relatively minor modifications of existing data that would enhance clarity.

Reviewer #1: Breadth of Screening (Line 103): The text states sera were screened against betacoronaviruses "including SARS-CoV-2 and MERS-CoV." This phrasing implies a broader panel (e.g., Lineage A). If only these two were tested, the text should be precise to avoid overstating the breadth.

**Response:** We thank the reviewer for this constructive suggestion to ensure the clarity of what we did. We have edited this sentence.

Lines 104-107: “To identify bnAbs targeting the conserved S2 stem helix of betacoronaviruses, we first screened sera from six SARS-CoV-2 convalescent donors (CC6, CC21, CC40, CC48, CC57, and CC65) for neutralizing activity against SARS-CoV-2 (sarbecovirus) and MERS-CoV (merbecovirus) pseudoviruses, which belong to different betacoronavirus lineages (Fig 1A-B).”

Figure 1E: SARS-CoV-1 and SARS-CoV-2 are represented by a single curve. While it is implied that they share an identical stem helix sequence, this should be explicitly stated in the figure legend.

**Response:** We apologize for not being clear enough for the description in the manuscript. Yes, SARS-CoV-1 and SARS-CoV-2 have the identical stem helix sequence as shown in Fig 2A. Among all the figure legends, SARS-CoV-1/2 first appeared in Fig 1D. So as per the reviewer’s suggestion, we added the following in the legend of Fig 1D.

Lines 403-404: “SARS-CoV-1/2 represents both SARS-CoV-1 and SARS-CoV-2 that have the same stem helix sequence.”

**Reviewer #2**: In this article titled “Directed evolution of a stem-helix–targeting antibody enables MERS-CoV crossneutralization through enhanced binding affinity” the authors Zhou, Yuan, Zhang, Limbo et al. describe the in vitro affinity maturation of an antibody to gain neutralization activity against MERS-CoV. Starting from an antibody that bound to the MERS-CoV stem helix region but did not neutralize the virus, the authors performed directed evolution targeting the antibody CDRs coupled with high throughput screening with antigen to obtain an antibody that was able to neutralize MERS-CoV, while also retaining neutralizing activity against other betacoronaviruses. Crystal structures in complex with antigen were determined for both the starting and the engineered antibody enabling high resolution understanding of the binding mode of the antibody as well as the impact of the mutations in the evolved antibody. Overall, this study reports important data and highlights how engineering existing antibodies can allow expansion of neutralization breadth – this is of value for developing broad reactivity to Coronaviruses and responding to future pandemics.

I am in support of publication of this manuscript in Plos Pathogens once the authors have had a chance to address the following:

**Response:** We thank the reviewer for their recognition and support of our work! Please see our detailed responses below for the comments/questions raised.

1. The CC65 serum neutralizes MERS-Cov (Figure 1A), yet the antibodies isolated from the serum do not neutralize MERS-CoV, which likely means there is a yet undiscovered antibody (or antibodies) in the serum that neutralizes MERS-CoV. Could the authors comment on this in the discussion?

**Response:** Yes, we couldn't agree more. In this paper, the single B cell sorting was performed using HCoV-HKU1 and SARS-CoV-2 S-2P proteins as baits. When the sorting was done with both SARS-CoV-2 and MERS-CoV S-2P proteins, it was possible to get some stem helix antibodies that had both MERS-CoV- and SARS-CoV-2-neutralizing activity, as we reported in our *Immunity* paper (https://doi.org/10.1016/j.immuni.2023.02.005). Unfortunately, we have limited amount of PBMCs from the CC65 donor, so that we couldn’t try another sorting strategy. Although monoclonal antibodies with both MERS-CoV- and SARS-CoV-2-neutralizing activity were not obtained from CC65 donor, CC65.1, which possesses MERS-CoV-stem-helix binding but not neutralization activity, provides us an ideal template to test if the neutralization breadth of stem helix antibodies can be expanded by in vitro affinity maturation. As per the reviewer’s suggestion, we have added some comments in the discussion.

Lines 343-348: “In this study, we show that sera from a SARS-CoV-2 convalescent donor (CC65) exhibit neutralizing activity against both SARS-CoV-2 and MERS-CoV, suggesting the presence of antibodies with dual neutralizing capacity. However, such antibodies were not isolated, likely due to the sorting strategy using SARS-CoV-2 and HCoV-HKU1 S-2P proteins as baits, rather than SARS-CoV-2 and MERS-CoV S-2P proteins as in our previous study. Notwithstanding, a MERS-CoV stem-helix-binding antibody, CC65.1, was obtained.”

2. Lines 174-175: “To confirm these epitopes recognized by CC65.1 and verify whether these residues are crucial for CC65.1 neutralization,…” The crystal structure has already confirmed that these epitopes are recognized by the antibody. The mutagenesis and binding/neutralization assays provides functional context to the observed contacts. I recommend revising the sentence.

**Response:** We thank the reviewer for this suggestion. We have revised this sentence.

Lines 179-181: “To verify the crucial residues for CC65.1 binding and neutralizing activity, we performed BLI binding and neutralization assays of CC65.1 to alanine scanning mutants of the SARS-CoV-2 stem helix peptide and pseudovirus, respectively.”

3. The affinity measurements reported are in the context of avidity because of the multivalent analytes and the authors appropriately designate these as apparent affinities. While not as rigorous as reporting intrinsic affinities using Fabs as analyte, the measurements reported seem sufficient for the goals of this study. Recommend adding a sentence clarifying the use of apparent affinity.

**Response:** We appreciate the reviewer’s comment. We agree that the binding measurements reported in this study reflect apparent affinities influenced by avidity effects, due to the multivalent nature of the IgG antibodies. As suggested, we have added the following clarifying sentence under the “BioLayer Interferometry binding (BLI)” section of “Materials and methods” indicating that the reported values represent apparent affinities rather than intrinsic monovalent binding affinities. We believe that these measurements are appropriate for comparing antibody binding in the context of this study.

Lines 660-663: “Different from intrinsic monovalent binding affinity of Fab, the IgG:spike or peptide binding can be a mixed population of 2:1 and 1:1 due to the multivalent nature of the IgG antibodies. Therefore, the term “apparent affinity” dissociation constants (KD^App^) are used to reflect the binding affinity between IgG antibodies and spike trimers or peptides tested.”

4. Figure 3 C and D: There is no description in the figure or in the figure legend for what the pink, yellow and orange dots are. Assume they are the antibodies shown in S6C. I recommend color coding the antibody names in Figure S6C, and using these colors in Figure 3 C and D (and in 3F), ie., use discrete colors for each variant.

**Response:** We are sorry for any difficulty this may have caused while reading. As per the reviewer’s suggestion, we tried to color the antibody names in Fig S6C and used the same colors in Fig 3C, 3D, 3F and S6E. But we found that if we only used colors to distinguish them, it becomes too busy and difficult to tell things apart. Additionally, there are a limited number of high-contrast colors available.

Finally, we decided to employ both shapes and colors to distinguish different antibodies in Fig 3C, 3D, 3F and S6E, and describe in the figure legends. We think this solution seems to be clearer. Please see the revised figures and figure legends.

**Fig 3C and 3D**

Lines 453-456, Fig 3: “(**C-D**) Comparison of the “apparent affinity” dissociation constants (KD^App^) of S2P6, parental CC65.1 and selected eCC65.1 mAbs against S-2P proteins and stem helix peptides from MERS-CoV (C) and SARS-CoV-2 (D), different antibodies are shown in assorted shapes and colors.”

**Fig 3F**

Lines 458-461, Fig 3: “(**F**) Plots showing correlation between KD^APP^ of S2P6, CC65.1 and selected eCC65.1 mAbs to MERS-CoV and SARS-CoV-2 stem helix peptides with neutralization potency (IC_50_) against their corresponding pseudoviruses. The same shapes and colors are employed to indicate different antibodies as in panel C.”

**Fig S6E**

Lines 1052-1055, Fig S6: “(**E**) Correlation analysis between KD^APP^ values of S2P6, CC65.1 and selected eCC65.1 mAbs for MERS-CoV and SARS-CoV-2 S-2P proteins and neutralization potency (IC_50_) against their corresponding pseudoviruses. Different antibodies are shown in assorted shapes and colors.”

5. BLI sensorgrams should be shown in a Supplemental figure for S6C.

**Response:** We thank the reviewer for this suggestion. We now show all the BLI curves related to the binding data shown in Fig S6C in Fig S7. Please see the following figure and figure legends.

**Fig S7**

Lines 1059-1064: **“Fig S7. BLI binding curves of S2P6, parental CC65.1 and selected eCC65.1 mAbs to spike proteins and stem helix peptides of SARS-CoV-2 and MERS-CoV**. All BLI binding curves related to the binding data of antibodies to S-2P proteins (**A-B**) and stem helix peptides (**C-D**) of SARS-CoV-2 and MERS-CoV shown in Fig S6C are displayed here. The kinetic curves were fit to a 1:1 binding model. S2P6 was used as a positive control. Different antibodies are shown in assorted lines and colors.”

**Minor:**

1. Lines 108-109: “Except stem helix region, sequence identify between SARS-CoV-2 and HCoV-HKU1 (embecovirus) spike is low.” Change “identify” to “identity”.

**Response:** We thank the reviewer for pointing out this typo. We have changed.

Lines 110-111: “Except for the stem helix region, sequence identity between the SARS-CoV-2 and HCoV-HKU1 (embecovirus) spikes is low.”
