# Supplementary material for: Directed evolution of a stem-helix–targeting antibody enables MERS-CoV cross-neutralization through enhanced binding affinity
Source: PLoS Pathog. 2026 Aug 7;22(8):e1014393. doi: 10.1371/journal.ppat.1014393 (PMC13480645; doi:10.1371/journal.ppat.1014393)
Supplement: Peer review history

# Peer review: Decision Letter 0

**DOI:** 10.1371/journal.ppat.1014393.r001

**Authors:** Ronald Swanstrom, Mario Borgnia

19 Feb 2026

PPATHOGENS-D-25-02460

Directed evolution of a stem-helix–targeting antibody enables MERS-CoV cross-neutralization through enhanced binding affinity

PLOS Pathogens

Dear Dr. Andrabi,

Thank you for submitting your manuscript to PLOS Pathogens. After careful consideration, we feel that it has merit but does not fully meet PLOS Pathogens's publication criteria as it currently stands. Therefore, we invite you to submit a revised version of the manuscript that addresses the points raised during the review process.

Please submit your revised manuscript by Apr 20 2026 11:59PM. If you will need more time than this to complete your revisions, please reply to this message or contact the journal office at plospathogens@plos.org. When you're ready to submit your revision, log on to https://www.editorialmanager.com/ppathogens/ and select the 'Submissions Needing Revision' folder to locate your manuscript file.

Please include the following items when submitting your revised manuscript:

* A letter that responds to each point raised by the editor and reviewer(s). You should upload this letter as a separate file labeled 'Response to Reviewers'. This file does not need to include responses to any formatting updates and technical items listed in the 'Journal Requirements' section below.

* A marked-up copy of your manuscript that highlights changes made to the original version. You should upload this as a separate file labeled 'Revised Manuscript with Track Changes'.

* An unmarked version of your revised paper without tracked changes. You should upload this as a separate file labeled 'Manuscript'.

If you would like to make changes to your financial disclosure, competing interests statement, or data availability statement, please make these updates within the submission form at the time of resubmission. Guidelines for resubmitting your figure files are available below the reviewer comments at the end of this letter.

We look forward to receiving your revised manuscript.

Kind regards,

Mario Juan Borgnia, Ph.D.

Guest Editor

PLOS Pathogens

Ronald Swanstrom

Section Editor

PLOS Pathogens

Sumita Bhaduri-McIntosh

Editor-in-Chief

PLOS Pathogens

orcid.org/0000-0003-2946-9497

Michael Malim

Editor-in-Chief

PLOS Pathogens

orcid.org/0000-0002-7699-2064

**Journal Requirements:**

1) Please ensure that the CRediT author contributions listed for every co-author are completed accurately and in full.

At this stage, the following Authors/Authors require contributions: Panpan Zhou, Meng Yuan, Yuexiu Zhang, Oliver Limbo, Ge Song, Fangzhu Zhao, Hejun Liu, Wan-ting He, Tazio Capozzola, Sean Callaghan, Gabriel Avillion, Xuduo Li, Nathan Beutler, Peter Yong, Fabio Anzanello, Thomas F Rogers, Dennis R Burton, Joseph G Jardine, Ian A Wilson, and Raiees Andrabi. Please ensure that the full contributions of each author are acknowledged in the "Add/Edit/Remove Authors" section of our submission form.

The list of CRediT author contributions may be found here: https://journals.plos.org/plospathogens/s/authorship#loc-author-contributions

2) We ask that a manuscript source file is provided at Revision. Please upload your manuscript file as a .doc, .docx, .rtf or .tex. If you are providing a .tex file, please upload it under the item type u2018LaTeX Source Fileu2019 and leave your .pdf version as the item type u2018Manuscriptu2019.

3) Please upload all main figures as separate Figure files in .tif or .eps format. For more information about how to convert and format your figure files please see our guidelines:

https://journals.plos.org/plospathogens/s/figures

4) We notice that your supplementary Figures, and Tables are included in the manuscript file. Please remove them and upload them with the file type 'Supporting Information'. Please ensure that each Supporting Information file has a legend listed in the manuscript after the references list.

5) We note that your Data Availability Statement is currently as follows: "All data associated with this study are present in the paper or the supplementary materials. The structures reported in this study are available under the accession codes PDB ID XXXX, XXXX and XXXX.". Please confirm at this time whether or not your submission contains all raw data required to replicate the results of your study. Authors must share the “minimal data set” for their submission. PLOS defines the minimal data set to consist of the data required to replicate all study findings reported in the article, as well as related metadata and methods (https://journals.plos.org/plosone/s/data-availability#loc-minimal-data-set-definition).

For example, authors should submit the following data:

1) The values behind the means, standard deviations and other measures reported;

2) The values used to build graphs;

3) The points extracted from images for analysis..

Authors do not need to submit their entire data set if only a portion of the data was used in the reported study.

If your submission does not contain these data, please either upload them as Supporting Information files or deposit them to a stable, public repository and provide us with the relevant URLs, DOIs, or accession numbers. For a list of recommended repositories, please see https://journals.plos.org/plosone/s/recommended-repositories.

If there are ethical or legal restrictions on sharing a de-identified data set, please explain them in detail (e.g., data contain potentially sensitive information, data are owned by a third-party organization, etc.) and who has imposed them (e.g., an ethics committee). Please also provide contact information for a data access committee, ethics committee, or other institutional body to which data requests may be sent. If data are owned by a third party, please indicate how others may request data access.

6) Please amend your detailed Financial Disclosure statement. This is published with the article. It must therefore be completed in full sentences and contain the exact wording you wish to be published.

- State the initials, alongside each funding source, of each author to receive each grant. For example: "This work was supported by the National Institutes of Health (####### to AM; ###### to CJ) and the National Science Foundation (###### to AM)."

- State what role the funders took in the study. If the funders had no role in your study, please state: "The funders had no role in study design, data collection and analysis, decision to publish, or preparation of the manuscript.".

If you did not receive any funding for this study, please simply state: u201cThe authors received no specific funding for this work.u201d

**Reviewers' Comments:**

Reviewer's Responses to Questions

**Part I - Summary**

Please use this section to discuss strengths/weaknesses of study, novelty/significance, general execution and scholarship.

Reviewer #1: The authors describe the isolation of CC65.1, a stem-helix targeting antibody, and its subsequent affinity maturation to generate variants (e.g., eCC65.1) capable of neutralizing MERS-CoV. The study is well-conceived, and the use of directed evolution to broaden the neutralizing breadth of antibodies targeting cryptic epitopes is of high interest to the field. The manuscript is well-written, and the structural analyses provided are robust. However, a few technical details regarding the spike constructs and mechanism of action need clarification to ensure a comprehensive understanding of the discovery.

Reviewer #2: In this article titled “Directed evolution of a stem-helix–targeting antibody enables MERS-CoV crossneutralization through enhanced binding affinity” the authors Zhou, Yuan, Zhang, Limbo et al. describe the in vitro affinity maturation of an antibody to gain neutralization activity against MERS-CoV. Starting from an antibody that bound to the MERS-CoV stem helix region but did not neutralize the virus, the authors performed directed evolution targeting the antibody CDRs coupled with high throughput screening with antigen to obtain an antibody that was able to neutralize MERS-CoV, while also retaining neutralizing activity against other betacoronaviruses. Crystal structures in complex with antigen were determined for both the starting and the engineered antibody enabling high resolution understanding of the binding mode of the antibody as well as the impact of the mutations in the evolved antibody. Overall this study reports important data and highlights how engineering existing antibodies can allow expansion of neutralization breadth – this is of value for developing broad reactivity to Coronaviruses and responding to future pandemics.

I am in support of publication of this manuscript in Plos Pathogens once the authors have had a chance to address the following:

1. The CC65 serum neutralizes MERS-Cov (Figure 1A), yet the antibodies isolated from the serum do not neutralize MERS-CoV, which likely means there is a yet undiscovered antibody (or antibodies) in the serum that neutralizes MERS-CoV. Could the authors comment on this in the discussion?

2. Lines 174-175: “To confirm these epitopes recognized by CC65.1 and verify whether these residues are crucial for CC65.1 neutralization,…” The crystal structure has already confirmed that these epitopes are recognized by the antibody. The mutagenesis and binding/neutralization assays provides functional context to the observed contacts. I recommend revising the sentence.

3. The affinity measurements reported are in the context of avidity because of the multivalent analytes and the authors appropriately designate these as apparent affinities. While not as rigorous as reporting intrinsic affinities using Fabs as analyte, the measurements reported seem sufficient for the goals of this study. Recommend adding a sentence clarifying the use of apparent affinity.

4. Figure 3 C and D: There is no description in the figure or in the figure legend for what the pink, yellow and orange dots are. Assume they are the antibodies shown in S6C. I recommend color coding the antibody names in Figure S6C, and using these colors in Figure 3 C and D (and in 3F), ie., use discrete colors for each variant.

5. BLI sensorgrams should be shown in a Supplemental figure for S6C.

Minor:

1. Lines 108-109: “Except stem helix region, sequence identify between SARS-CoV-2 and HCoV-HKU1 (embecovirus) spike is low.” Change “identify” to “identity”.

**********

**Part II – Major Issues: Key Experiments Required for Acceptance**

Please use this section to detail the key new experiments or modifications of existing experiments that should be absolutely required to validate study conclusions.

Generally, there should be no more than 3 such required experiments or major modifications for a "Major Revision" recommendation. If more than 3 experiments are necessary to validate the study conclusions, then you are encouraged to recommend "Reject".

Reviewer #1: 1. Mechanism of Neutralization: The manuscript lacks a discussion on how affinity maturation enables neutralization of MERS-CoV when the parent antibody binds but does not neutralize. The current explanation treats affinity as a standalone metric. The authors should explicitly connect their hypothesis on accessibility (Lines 258-260) to the neutralization data. Does the high affinity of eCC65.1 allow it to "catch" the transiently exposed stem helix on MERS-CoV more effectively, thereby increasing occupancy and blocking the hairpin formation in the fusion machinery? This complexity of epitope accessibility is further highlighted by the SARS-CoV-1 data. Lines 121-122 claim "stronger binding" for SARS-CoV-1 in "both formats," yet CELISA data (Fig S1B) shows significantly weaker binding for SARS-CoV-1 compared to SARS-CoV-2, despite identical epitope sequences. Discussing this "occupancy model" is necessary to resolve the apparent paradox of why the parent antibody binds but fails to neutralize.

2. Clarification of Spike Constructs: The text fails to explicitly state whether WT, S-2P, or HexaPro constructs were used for the specific spike proteins (SARS-CoV-1/2 vs. MERS-CoV) in the binding assays (SPR/BLI). Please provide these details in the Methods section.

3. Terminology: The authors frequently use the term "rational engineering." However, the approach utilized is directed evolution, not rational design. In a typical rational design approach (e.g., structure-guided), one would target the direct interface residues to improve affinity. In contrast, the key impact here appears to come from paratope stabilization between CDRL1 and L2, potentially reducing the entropic cost of capturing a transiently breathing epitope. Notably, the interacting residues are effectively the same (with the exception of the S93A mutation), while the significant improvements arise from distal stabilization (e.g., the Lys-His interaction stabilizing the Tyr rotamer). I recommend reframing the text to highlight that directed evolution was capable of identifying this sophisticated, non-intuitive structural solution, emphasizing the power of the method used.

Reviewer #2: (No Response)

**********

**Part III – Minor Issues: Editorial and Data Presentation Modifications**

Please use this section for editorial suggestions as well as relatively minor modifications of existing data that would enhance clarity.

Reviewer #1: Breadth of Screening (Line 103): The text states sera were screened against betacoronaviruses "including SARS-CoV-2 and MERS-CoV." This phrasing implies a broader panel (e.g., Lineage A). If only these two were tested, the text should be precise to avoid overstating the breadth.

Figure 1E: SARS-CoV-1 and SARS-CoV-2 are represented by a single curve. While it is implied that they share an identical stem helix sequence, this should be explicitly stated in the figure legend.

Reviewer #2: (No Response)

**********

PLOS authors have the option to publish the peer review history of their article (what does this mean?). If published, this will include your full peer review and any attached files.

If you choose “no”, your identity will remain anonymous but your review may still be made public.

**Do you want your identity to be public for this peer review?**  For information about this choice, including consent withdrawal, please see our Privacy Policy.

Reviewer #1: **Yes:** Arvind Sharma

Reviewer #2: No

[NOTE: If reviewer comments were submitted as an attachment file, they will be attached to this email and accessible via the submission site. Please log into your account, locate the manuscript record, and check for the action link "View Attachments". If this link does not appear, there are no attachment files.]

**Figure resubmission:**

**Reproducibility:**

To enhance the reproducibility of your results, we recommend that authors of applicable studies deposit laboratory protocols in protocols.io, where a protocol can be assigned its own identifier (DOI) such that it can be cited independently in the future. Additionally, PLOS ONE offers an option to publish peer-reviewed clinical study protocols. Read more information on sharing protocols at https://plos.org/protocols?utm_medium=editorial-email&utm_source=authorletters&utm_campaign=protocols

# Author comment: Author response to Decision Letter 1

**DOI:** 10.1371/journal.ppat.1014393.r002

30 Apr 2026

Attachment Submitted filename: Response to Reviewers.docx

# Peer review: Decision Letter 1

**DOI:** 10.1371/journal.ppat.1014393.r003

**Authors:** Ronald Swanstrom, Mario Borgnia

22 Jun 2026

Dear Dr Andrabi,

We are pleased to inform you that your manuscript 'Directed evolution of a stem-helix–targeting antibody enables MERS-CoV cross-neutralization through enhanced binding affinity' has been provisionally accepted for publication in PLOS Pathogens.

Before your manuscript can be formally accepted you will need to complete some formatting changes, which you will receive in a follow up email. A member of our team will be in touch with a set of requests.

Please note that your manuscript will not be scheduled for publication until you have made the required changes, so a swift response is appreciated.

IMPORTANT: The editorial review process is now complete. PLOS will only permit corrections to spelling, formatting or significant scientific errors from this point onwards. Requests for major changes, or any which affect the scientific understanding of your work, will cause delays to the publication date of your manuscript.

Should you, your institution's press office or the journal office choose to press release your paper, you will automatically be opted out of early publication. We ask that you notify us now if you or your institution is planning to press release the article. All press must be co-ordinated with PLOS.

Thank you again for supporting Open Access publishing; we are looking forward to publishing your work in PLOS Pathogens.

Best regards,

Mario Juan Borgnia, Ph.D.

Guest Editor

PLOS Pathogens

Ronald Swanstrom

Section Editor

PLOS Pathogens

Sumita Bhaduri-McIntosh

Editor-in-Chief

PLOS Pathogens

orcid.org/0000-0003-2946-9497

Michael Malim

Editor-in-Chief

PLOS Pathogens

orcid.org/0000-0002-7699-2064

***********************************************************

Reviewer Comments (if any, and for reference):

Reviewer's Responses to Questions

**Part I - Summary**

Please use this section to discuss strengths/weaknesses of study, novelty/significance, general execution and scholarship.

Reviewer #1: (No Response)

Reviewer #2: The authors have adequately responded to reviewer critiques. I recommend publication of this manuscript.

**********

**Part II – Major Issues: Key Experiments Required for Acceptance**

Please use this section to detail the key new experiments or modifications of existing experiments that should be absolutely required to validate study conclusions.

Generally, there should be no more than 3 such required experiments or major modifications for a "Major Revision" recommendation. If more than 3 experiments are necessary to validate the study conclusions, then you are encouraged to recommend "Reject".

Reviewer #1: (No Response)

Reviewer #2: (No Response)

**********

**Part III – Minor Issues: Editorial and Data Presentation Modifications**

Please use this section for editorial suggestions as well as relatively minor modifications of existing data that would enhance clarity.

Reviewer #1: (No Response)

Reviewer #2: (No Response)

**********

PLOS authors have the option to publish the peer review history of their article (what does this mean?). If published, this will include your full peer review and any attached files.

If you choose “no”, your identity will remain anonymous but your review may still be made public.

**Do you want your identity to be public for this peer review?**  For information about this choice, including consent withdrawal, please see our Privacy Policy.

Reviewer #1: **Yes:** arvind sharma

Reviewer #2: No

# Editor report: Acceptance letter

**DOI:** 10.1371/journal.ppat.1014393.r004

**Authors:** Ronald Swanstrom, Mario Borgnia

Dear Dr Andrabi,

We are delighted to inform you that your manuscript, "Directed evolution of a stem-helix–targeting antibody enables MERS-CoV cross-neutralization through enhanced binding affinity," has been formally accepted for publication in PLOS Pathogens.

We have now passed your article onto the PLOS Production Department who will complete the rest of the pre-publication process. All authors will receive a confirmation email upon publication.

The corresponding author will soon be receiving a typeset proof for review, to ensure errors have not been introduced during production. Please review the PDF proof of your manuscript carefully, as this is the last chance to correct any scientific or type-setting errors. Please note that major changes, or those which affect the scientific understanding of the work, will likely cause delays to the publication date of your manuscript. Note: Proofs for Front Matter articles (Pearls, Reviews, Opinions, etc...) are generated on a different schedule and may not be made available as quickly.

Soon after your final files are uploaded, the early version of your manuscript, if you opted to have an early version of your article, will be published online. The date of the early version will be your article's publication date. The final article will be published to the same URL, and all versions of the paper will be accessible to readers.

For Research Articles, you will receive an invoice from PLOS for your publication fee after your manuscript has reached the completed accept phase. If you receive an email requesting payment before acceptance or for any other service, this may be a phishing scheme. Learn how to identify phishing emails and protect your accounts at https://explore.plos.org/phishing.

Thank you again for supporting open-access publishing; we are looking forward to publishing your work in PLOS Pathogens.

Best regards,

Sumita Bhaduri-McIntosh

Editor-in-Chief

PLOS Pathogens

orcid.org/0000-0003-2946-9497

Michael Malim

Editor-in-Chief

PLOS Pathogens

orcid.org/0000-0002-7699-2064
